# The molecular heterogeneity of the precancerous breast affects drug efficacy

**DOI:** 10.1038/s41598-022-16779-y

**Published:** 2022-07-22

**Authors:** Anjana Bhardwaj, Raniv Dawey Rojo, Zhenlin Ju, Alexander Koh, Kazunoshin Tachibana, Jing Wang, Isabelle Bedrosian

**Affiliations:** 1 Department of Breast Surgical Oncology, The University of Texas MD Anderson Cancer Center, Houston, TX 77030 USA; 2 Department of Bioinformatics and Computational Biology, The University of Texas MD Anderson Cancer Center, Houston, TX 77030 USA; 3 Present Address: College of Medicine, University of the Philippines Manila, Quezon City, Philippines; 4 Present Address: Department of Breast Surgery, Fukushima Medical University School of Medicine, Fukushima, Japan

**Keywords:** Mechanisms of disease, Cancer prevention, Breast cancer

## Abstract

In the therapeutic domain, targeted therapies have been shown to be generally more effective when given to patients with tumors that harbor the targeted aberration. This principle has not been tested in cancer prevention despite evidence that molecular heterogeneity accompanies the multi-step progression to invasive disease. We hypothesized that efficacy of agents targeting the precancerous state varies based on timing of the treatment relative to the underlying molecular changes. MCF10A cell line-based model of the multi-step progression to TNBC was used. Global proteomic patterns were obtained and growth-inhibitory effects of selected agents were correlated with the underlying molecular stage of progression. These analyses revealed that most protein alterations were acquired in the normal-to-atypia (preneoplasia) transition, with only handful aberrations acquired hereafter. The efficacy of small molecule inhibitors of the AKT/MEK pathway was associated with the underlying pathway levels. Similarly, fluvastatin was more effective in inhibiting cell proliferation earlier in the progression model. However, the nonspecific inhibitors, aspirin and metformin, were equally ineffective in inhibiting proliferation across the progression model. Our data provides proof-of-principle that in the prevention domain, treatment with agents developed to target specific pathways, will need to consider the molecular heterogeneity of the precancerous breast in order to achieve maximum efficacy.

## Introduction

Over the last two decades, aided by breakthroughs in genomic profiling of tumors, the therapeutic approach to treatment of cancer has increasingly shifted from use of unselected chemotherapy agents to more specific targeted agents. Such targeted therapies have, in general, been shown to be more effective when given to patients whose tumors harbor the underlying pathway mutation as compared to unselected cohorts suggesting the cancer driver role of these aberrations^1,2^. This principle of tailoring therapy to the underlying tumor biology has not, however, been extended to the domain of prevention. For instance, tamoxifen (an estrogen modulator) is used as a cancer prevention agent with out understanding if the woman is at risk of developing breast cancer that is driven by estrogen receptor ER or not. This is in part because of the paucity of agents available for prevention, but also because of limited understanding of the sequential molecular alterations that accompany the lengthy, multi-step progression to invasive disease and their relavence to treatment efficacy.

We have previously reported that in a cell line-based model of breast cancer progression, the majority of transcriptomic and miRNAomic alterations occurred early during the transition from the normal to preneoplastic state^3–5^. Similarly, others have reported that a majority of miRNA and mRNA alterations, mutational burden and gene amplifications are acquired early during breast cancer development, underscoring the molecular diversity that exists within breast precancerous state^6,7^. Understanding the timeline of this molecular heterogeneity, including proteomic alterations, during breast cancer progression and how it affects the efficacy of drugs that target these aberrations is important for designing effective chemopreventive regimens. The goal of the present study was to describe a global view of proteomic alterations that occur during the stepwise progression of breast cancer and to investigate the association between protein dysregulation at various precancer stages of breast tumorigenesis and efficacy of drugs for breast cancer prevention. To test this association, as a proof of concept, targeted therapies, as well as non-specific therapies that have been used for breast cancer prevention and treatment, were tested in an MCF10A -based model of breast cancer progression.

## Methods

### Cell lines

We used a panel of MCF10A isogenic cell lines that represent a model of breast cancer progression generated at Karmanos Cancer Center^8,9^. This model includes the following cell lines: MCF10A (P)—a non-cancer normal-like mammary cell line, MCF10.NeoT and MCF10.AT1—that represent preneoplastic (benign) cell lines, MCF10.DCIS—a ductal carcinoma in situ cell line and MCF10.CA1D -an invasive breast cancer cell line. MCF10A(P) human mammary epithelial cells were obtained from ATCC and grown in a glutamine-fortified DMEM/F12 (50:50) medium. The growth medium was supplemented with horse serum (5%), insulin (10 μg/ ml), EGF (20 ng/ml), hydrocortisone (0.5 µg/ml) and cholera toxin (100 ng/ml). Preneoplastic (benign) MCF10.NeoT (hyperplasia) and MCF10.AT1 (atypical hyperplasia) cells were grown in glutamine-fortified DMEM/F12 (50:50) medium supplemented with CaCl_2_ (1.05 mM), horse serum (5%), HEPES (10 mM), insulin (10 μg/ ml), EGF (20 ng/ml) and hydrocortisone (0.5 µg/ml). Ductal carcinoma in situ (MCF10.DCIS) cells were obtained from DCIS.com and invasive MCF10.CA1D cells were obtained from Karmanos Cancer Center and grown in a glutamine-fortified DMEM/F12 (50:50) medium with CaCl_2_ (1.05 mM), horse serum (5%), and HEPES (10 mM). The cell lines were used within the initial 10 passages after purchase.

### RPPA analysis

Exponentially growing MCF10A panel cell lines were processed to extract whole cellular proteins for reverse phase protein array (RPPA) analysis as described elsewhere^10,11^. Briefly, the cells were washed with cold phosphate-buffered saline and scraped off the tissue culture dishes. The cells were pelleted by spinning at high speed at 4 °C. Next, the cell pellet was lysed in a dye-free whole-cell lysis buffer containing protease inhibitors. Protein concentration was measured, and 100 µg protein/cell line was sent for RPPA to the RPPA Core facility at MD Anderson Cancer Center. RPPA slides were quantified using Array-Pro (Media Cybernetics, Washington, DC) to generate spot signal intensities, which were then processed using the SuperCurve R package^12^ to estimate the relative protein expression level. The raw protein expression data were normalized by a set of loading controls as described before^10^. RPPA slide quality was assessed by a quality control classifier^11^, and only those slides with a value above 0.8 (range: 0–1) were processed for further analysis. Three separate replicates of each of these cell lines were used for RPPA analysis.

### MTT assay

Cell viability and proliferation were measured by MTT, a colorimetric assay as described elsewhere^13^. Briefly, cells were plated at a density of 5000 cells/well in 96-well tissue culture plates and were allowed to attach overnight prior to the start of treatment with either a PI3K inhibitor (LY294002), a MEK inhibitor (PD0325901), fluvastatin, metformin, aspirin or vehicle control. After 48-h treatment with these agents at the indicated concentrations, MTT agent was added and the cells were further incubated for about 2 h. The assay is based on the principle that viable cells metabolically reduce the MTT agent and form blue formazan crystals. This colorimetric reaction can be measured at 590 nm, after solubilizing the crystals with DMSO. A background absorbance at 630 nm was deducted from the MTT absorbance at 590 nm. Vehicle-treated wells with the highest absorbance were set as 100% cell proliferation, and the relative reduction in cell proliferation was calculated for each condition. The cell proliferation values (%) were computed to derive an IC50 value (the drug concentration that causes 50% reduction in cell proliferation) for each agent using GraphPad Prism software (version 9)^14^.

### Determining treatment efficacy

A combination of two separate criteria were used to designate a cell line as relatively sensitive: (i) if the IC50 was significantly lower (p < 0.05), and (ii) if the IC50 value was in the nanomolar to micromolar range, which would be consistent with dosing feasible for clinical use. Cell lines with IC50 values in the millimolar range were considered therapy resistant, and such drugs were considered poor inhibitors (or clinically irrelevant) as most therapeutic drugs achieve an average of about 10 µM concentration in blood plasma.

### Western blotting

Total cellular proteins from the various cells were subjected to polyacrylamide gel electrophoresis to analyze the levels of total and phosphorylated proteins (HMGCR, pAKT, AKT, pS6, S6, pMEK1/2, MEK1/2, AMPK, pAMPK, vinculin and β-actin) by Western blotting as described previously^15^. A single nitrocellulose membrane was probed with up to six antibodies (Cell Signalling Technologies) by first cutting the membrane into three parts that were probed with two primary antibodies, one raised in mouse and the other raised in rabbit, followed by a different fluorophore-labeled secondary antibody for detecting signal coming from both mouse and rabbit primary antibodies. An Odyssey imaging system (LiCOR) was used to capture the fluorescence signal and quantify the proteins. Vinculin and β-actin cytoskeleton proteins were used as internal controls for normalizing the amount of protein loaded. The same vinculin or β-actin bands may be shown for multiple proteins if they came from the same membrane. Phosphorylated proteins were measured by normalizing their signals with the respective total protein signals as well.

### Statistical analysis

Statistical significance was determined by Student’s *t* test, and p values < 0.05 were considered significant. MTT data were analyzed by one-way ANOVA followed by the Kruskal–Wallis test using GraphPad Prism software. RPPA data was analysed using one-way ANOVA. A post hoc test, Tukey's honestly significant difference (Tukey's HSD), was used to test pairwise differences while controlling the Type I errors. A false discovery rate (FDR) of < 5% was used as the significance level to select differentially expressed proteins by one-way ANOVA. In pairwise comparisons, significant proteins were identified by a Tukey’s adjusted p value of < 0.05 and fold change of at least 2. Two-way unsupervised heatmaps were used to display protein expression patterns. Pearson correlation was used to construct a correlation matrix and Ward’s linkage rule was used to cluster samples and proteins.

## Results

### Proteomic profiling reveals a dynamic landscape during TNBC tumorigenesis

**Figure 1:**
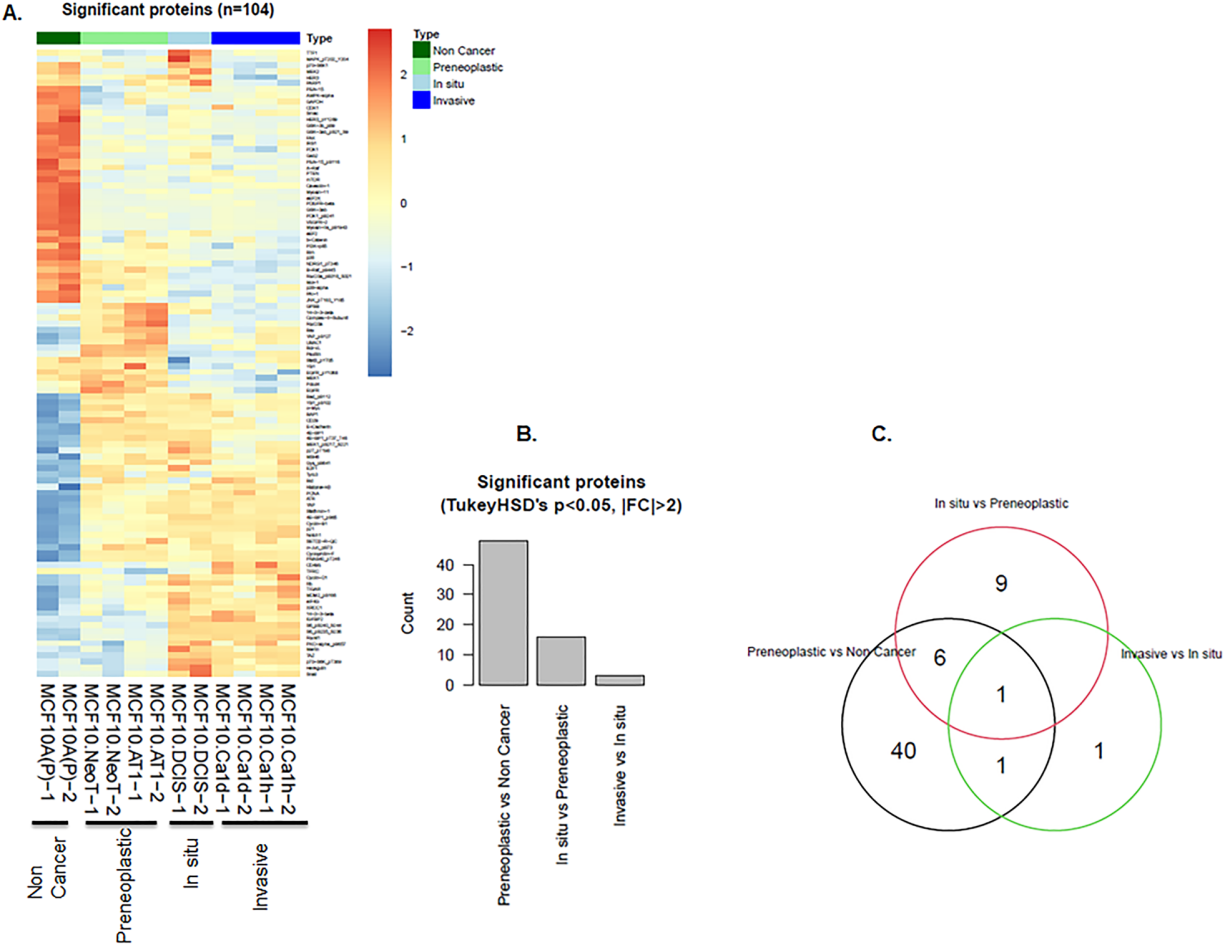
The majority of proteomic landscape changes during breast tumorigenesis in a triple-negative MCF10A cell line-based model occur in the transition from a normal to benign state. (**A**–**C**) Proteins that significantly changed during various stages of TNBC progression. (**A**) Heatmap generated from RPPA data shows an overview of alterations. Each row represents a specific protein’s highest expression (orange) or lowest expression (blue) in the various cell lines. Two replicates are shown for each cell line. (**B**) Bar diagram shows the number of proteins that changed for each transition from one stage of TNBC progression to another. (**C**) Venn diagram shows the number of deregulated proteins that overlapped between the indicated stage transitions.

The RPPA-based proteomic profiling of the MCF10A derived cell lines showed 217 proteins that changed during at least one of the transitions between stages of breast cancer progression (Fig. S1). Of these, 104 showed significant alteration (FDR < 5%; Fig. 1). The majority of protein aberrations occurred during the transition from a normal-like (non-cancer) state to a atypia (preneoplastic ) state (Fig. 1, Figs. S1, S12), with fewer alterations at subsequent transition points. Among the 104 significantly altered proteins, 48 proteins changed significantly between the normal-like and preneoplastic states (Fig. S2A), 16 proteins changed significantly between the preneoplastic and DCIS states (Fig. S2B) and 3 proteins changed significantly between the DCIS and invasive cancer states (Fig. S2C).

Of the total 16 proteins that significantly changed from preneoplastic to DCIS, we identified 6 proteins (FoxO3a_ pS318_S321, Bcl-xL, Cyclin-B1, MEK2, 4E-BP1_pS65 and S6_pS235_S236) that showed a pattern of continuous and significant change across the model system through to DCIS, the final targetable step for prevention of invasive disease (Fig. S2B). Of these 6 proteins, 2 proteins (Bcl-xL and FoxO3a_pS318_S321) had decreased expression during this multi-step transition, while the remaining 4 proteins, all of which were effector molecules of or fed in to the activation of pAKT-S6 and the MAPK pathway, had increased expression. None of these 6 proteins continued to change in the DCIS-to-invasive transition. Only 3 de novo protein alterations were identified in the transition from DCIS to the invasive state in our cell line model: PAI-1, NDRG1_pT346 and p70-S6K1 (Fig. S2C). It was interesting to note that although both MCF10.NeoT and MCF10.AT1 cell lines are considered preneoplastic based on histologic classification, they were not molecularly identical (Fig. S2A). This finding underscores i) the limitations of using histologic classification as a surrogate for molecular stage of progression and ii) the molecular heterogeneity of the preneoplastic state.

### Efficacy of targeted therapy is associated with baseline activation of target pathways during multi-step tumorigenesis

Two targetable oncogenic pathways that were found to be upregulated between the preneoplastic and DCIS states are : the MEK and pAKT-S6 pathways (MEK2 and S6_pS235_S236) (Fig. S2B). As proof of principle, and to test our hypothesis that the efficacy of targeted therapy in the prevention domain correlates with the levels of molecular alteration in the targeted pathway, we tested small molecular inhibitors of the MEK pathway (PD0325901) and the pAKT-pS6 pathway (PI3K inhibitor LY294002) on cell growth and proliferation in our multi-step tumorigenesis model.

**Figure 2:**
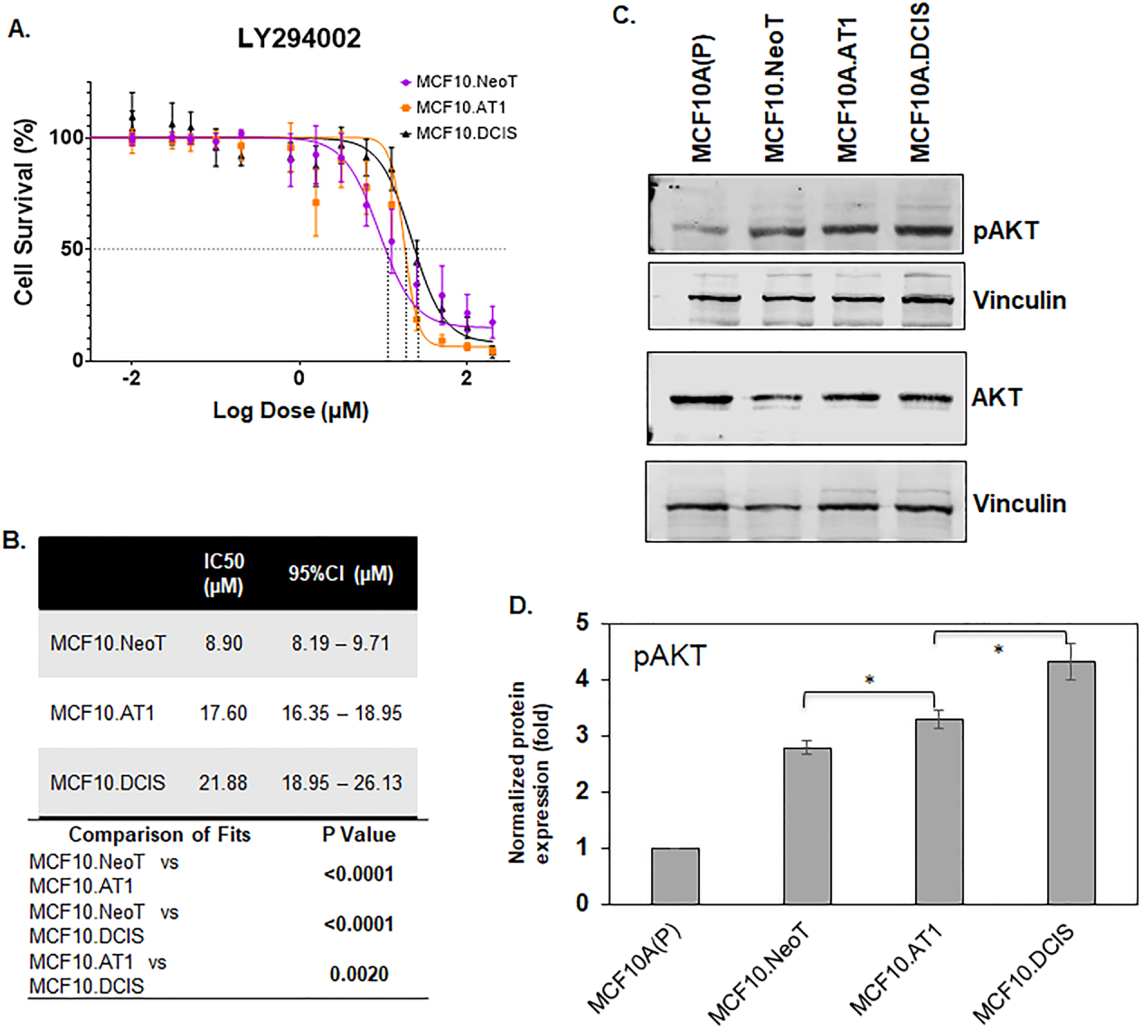
Aberrant activation of the AKT-mTOR pathway at baseline is associated with lower efficacy of a PI3K inhibitor. (**A**) Cell survival curves as measured by MTT assay show inhibition in proliferation of MCF10.NeoT, MCF10.AT1 and MCF10.DCIS cells by PI3K inhibitor LY294002. (**B**) Table shows the concentrations (in µM, with 95% confidence intervals [95%CI]) that cause 50% inhibition in cell survival (IC50) for each cell line and their statistical significance. A p value < 0.05 was considered statistically significant. (**C**) Western blot and (**D**) its quantification show upregulation of pAKT in the MCF10A breast cancer progression panel. pAKT expression was normalized to vinculin and total AKT levels. The same vinculin or β-actin bands may be shown for multiple proteins (and figures) if they came from the same membrane. Bar diagram: average values ± standard error of the mean (SEM). *p < 0.05.

An MTT assay was performed using the preneoplastic (MCF10.NeoT, MCF10.AT1) and DCIS (MCF10. DCIS) cell lines and cell proliferation at 48 h of drug treatment was used to determine IC50 of the agents tested. For the PI3K inhibitor LY294002, The IC50 was found to be the lowest in the MCF10.Neo T cells (8.90 µM), followed by the MCF10.AT1 cells (17.60 µM), with the highest IC50 value (21.88 µM) in the MCF10.DCIS cells (Fig. 2A,B). To understand the observed pattern of efficacy, the basal levels of pAKT, a downstream effector of the PI3K pathway, for each cell line were measured by Western blotting. We hypothesized that aberrant upregulation of pAKT levels during breast cancer tumorigenesis would inversely correlate with the efficacy of the PI3K inhibitor and positively correlate with the IC50 values. Consistent with this hypothesis, we found a gradual but significant increase in pAKT levels in MCF10.AT1 and MCF10.DCIS cells (19.7% and 54.8% induction, respectively; p < 0.05) relative to MCF10.NeoT cells (Fig. 2C,D, Figs. S5 and S6) that correlated with increases in IC50 values across this stepwise model.

**Figure 3:**
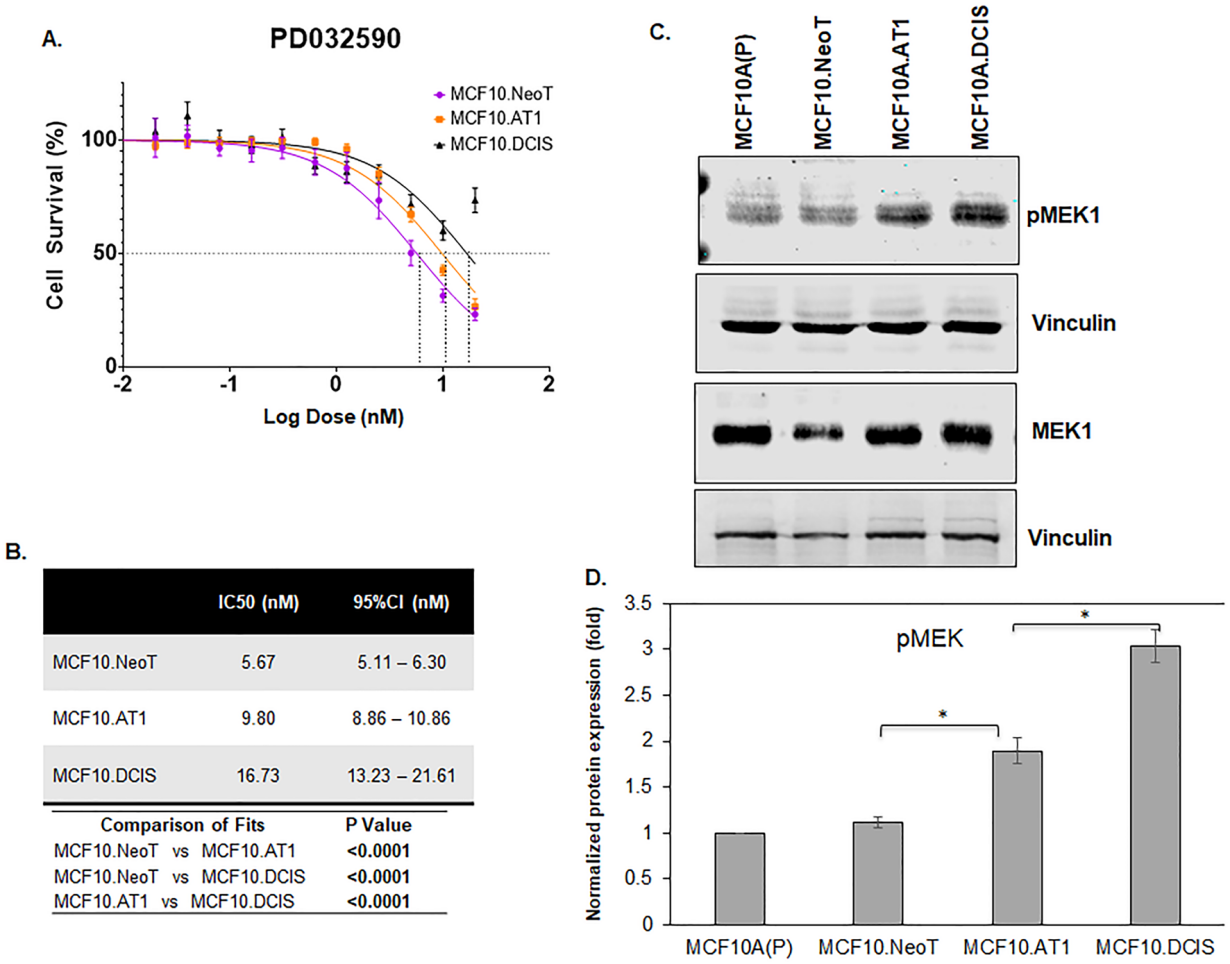
Aberrant activation of the pMEK pathway at baseline is associated with lower efficacy of a MEK inhibitor. (**A**) Cell survival curves as measured by MTT assay show inhibition in proliferation of MCF10.NeoT, MCF10.AT1 and MCF10.DCIS cells by MEK inhibitor PD0325901. (**B**) Table shows the IC50 and p values for each cell line. (**C**) Western blot and (**D**) its quantification shows upregulation of pMEK in the MCF10A breast cancer progression panel. pMEK expression was normalized to vinculin and total MEK levels. The same vinculin or β-actin bands may be shown for multiple proteins (and figures) if they came from the same membrane.Bar diagram: average values ± standard error of the mean (SEM). *p < 0.05.

Similarly, for the MEK inhibitor PD0325901, after 48 h of drug treatment, the IC50 value for PD0325901 was found to be lowest in the MCF10.NeoT (5.67 nM) cells, followed by 9.80 nM in MCF10.AT1 cells and 16.73 nM in the MCF10.DCIS cells (Fig. 3A,B). When the basal levels of pMEK were measured by Western blotting, we again observed a correlation between the efficacy as measured by IC50 values and the baseline activation of the pathway. The highest MEK efficacy (lowest IC50 value) was observed in the MCF10.NeoT cells, which had relatively low levels of MEK activation, and lower efficacy (higher IC50 values) was seen in later stages of the model, which had higher basal pMEK levels (1.7-fold and 2.7-fold in MCF10.AT1 and MCF10.DCIS cells, respectively, p < 0.05 relative to MCF10.NeoT cells) (Fig. 3C,D and Fig. S7). It is plausible that an upstream gene mutation led to the differential activation of the AKT and MEK pathways in precancer state vs DCIS state and thus determines the efficacy of pathway targeting by small molecule inhibitors (LY294002 and PD0325901) in MCF10.AT1 and MCF10.DCIS cells^16^. Prior studies have shown both the precancer MCF10.AT1 cells and DCIS cells to harbor same pathway activating mutations (H-Ras that activates MEK pathway and PIK3CA that activates AKT pathway)^8,17,18^. This potentially suggests that the observed differential drug efficacy of inhibitors (PI3K and MEK inhibitors) is independent of underlying pathway mutation and rather transcriptional and post-transcriptional mechanisms are likely to play a role in rendering the advanced cancer cells (DCIS) more aggressive and less responsive to therapies.

**Figure 4:**
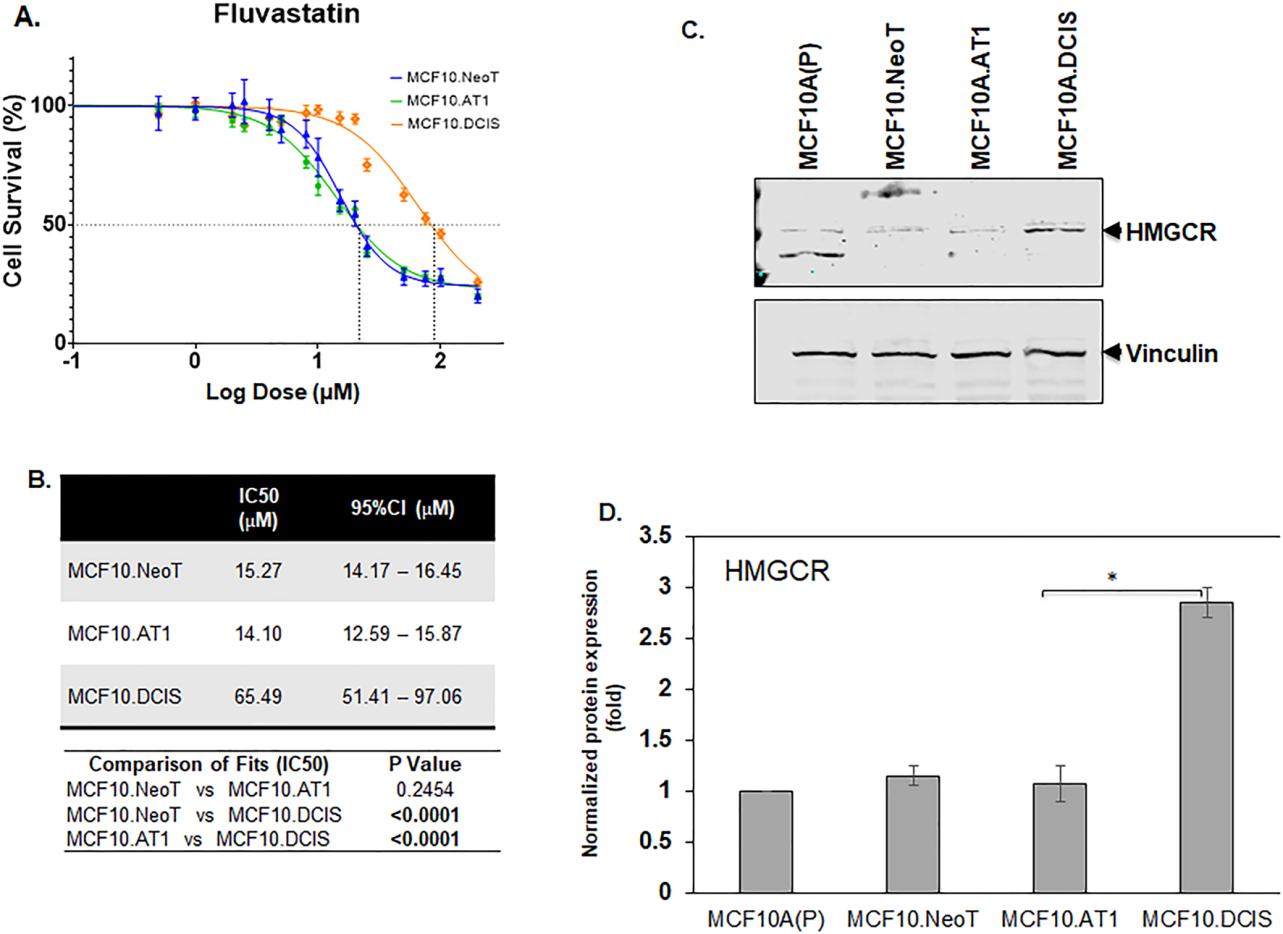
Aberrant activation of HMGCR at baseline is associated with lower efficacy of fluvastatin. (**A**) Cell survival curves as measured by MTT assay show inhibition in proliferation of MCF10.NeoT, MCF10.AT1 and MCF10.DCIS cells by a cholesterol-lowering drug, fluvastatin. (**B**) Table shows the IC50 and p values for each cell line. p < 0.05 was considered statistically significant. (**C**) Western blot and (**D**) its quantification shows upregulation of HMGCR in the MCF10A breast cancer progression panel. HMGCR expression was normalized to vinculin. Bar diagram: average values ± standard error of the mean (SEM). *p < 0.05.

In addition to these two pathways that were identified using RPPA, we have previously reported HMGCR, a rate-limiting enzyme in the cholesterol biosynthesis pathway, to be aberrantly upregulated in this breast cancer progression model^4^. Therefore, we also tested the efficacy of fluvastatin, a cholesterol-lowering drug that targets HMGCR activity, in this model system. As with the small molecule inhibitors, we found the IC50 value for fluvastatin to be the lowest in the preneoplastic cell lines, MCF10.NeoT and MCF10.AT1 (15.27 and 14.10 µM, respectively), compared to the MCF10.DCIS cells (65.49 µM) (Fig. 4A,B and Fig. S8), and this pattern correlated with an increasing basal level of HMGCR in DCIS cells (1.7-fold in MCF10.DCIS cells, p < 0.05 relative to MCF10.NeoT cells) (Fig. 4C,D). This finding was somewhat surprising; given that statins are reported to have pleiotropic effects^17,18^, we had anticipated that there would not be a strong correlation between HMGCR levels and efficacy in reducing cell growth and proliferation.

### Nonspecific drugs do not show context-specific efficacy

**Figure 5:**
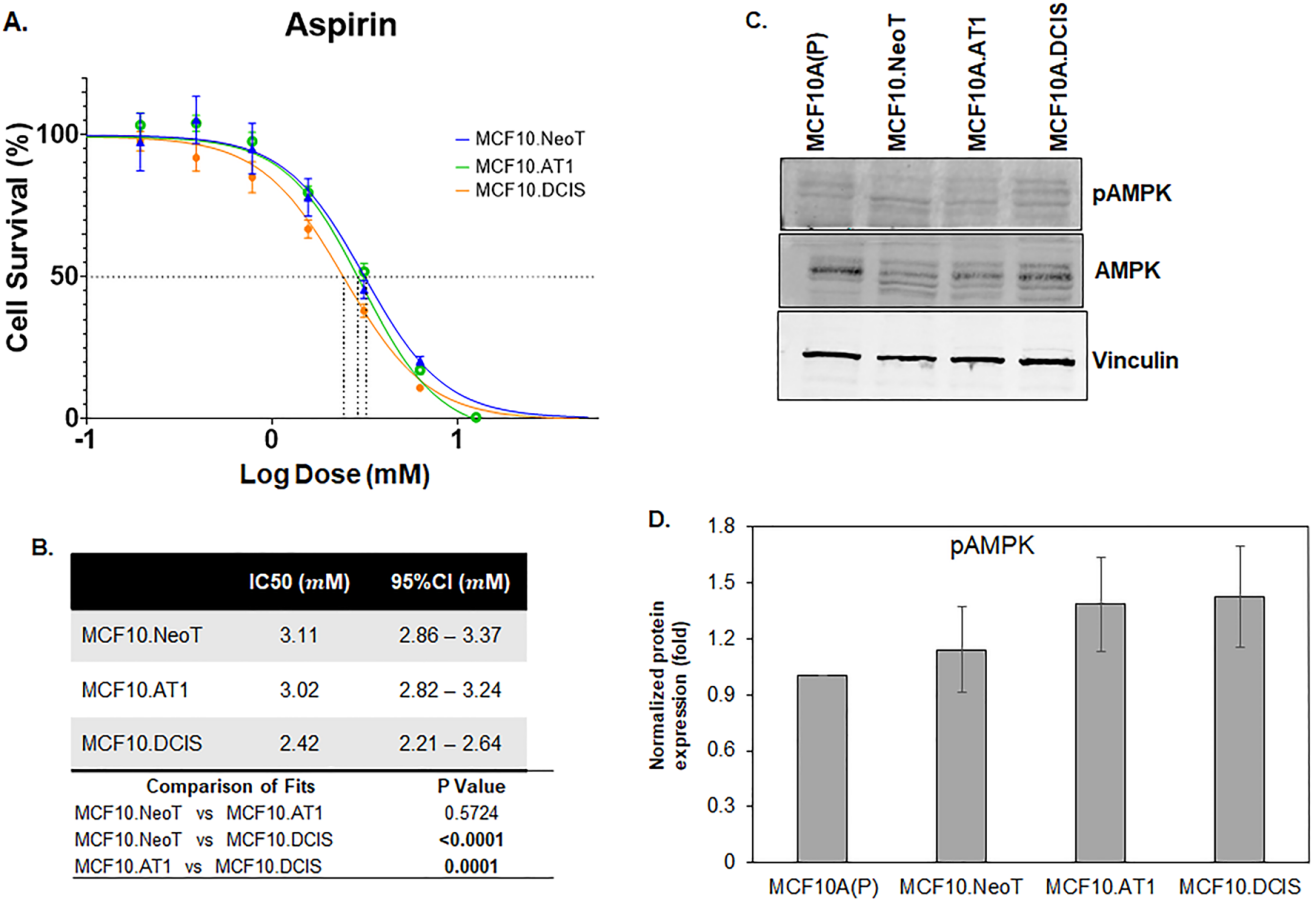
Baseline pAMPK levels do not change and are not associated with the efficacy of aspirin. (**A**) Cell survival curves as measured by MTT assay show inhibition in proliferation of MCF10.NeoT, MCF10.AT1 and MCF10.DCIS cells by aspirin, an AMPK activator. (**B**) Table shows the IC50 values (in mM) and p values for each cell line. (**C**) A Western blot and (**D**) its quantification shows pAMPK levels in the MCF10A breast cancer progression panel. Expression was normalized to vinculin. Bar diagram: average values ± standard error of the mean (SEM). *p < 0.05.

**Figure 6:**
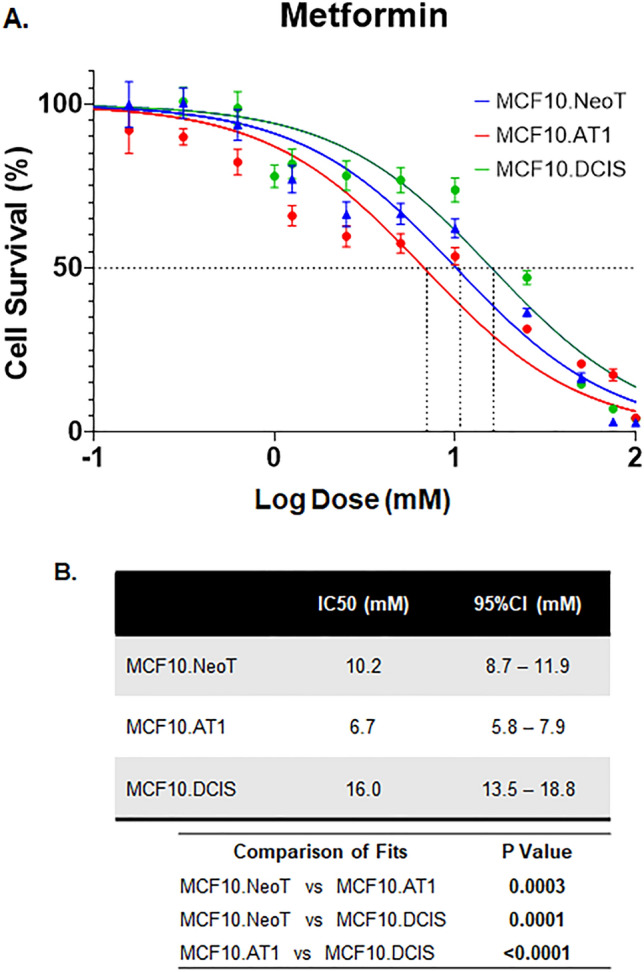
Efficacy of metformin chemoprevention in preneoplastic and DCIS cells. (**A**) Cell survival curves as measured by MTT assay show inhibition in proliferation of MCF10.NeoT, MCF10.AT1 and MCF10.DCIS cells by metformin, another AMPK activator. (**B**) Table shows the IC50 values (in mM) and p values for each cell line. pAMPK levels were shown in Fig. 5C,D.

Lastly, we tested two drugs that have been proposed for chemoprevention and do not have any direct oncogenic targets, aspirin and metformin^19,20^. These drugs have been tested in chemoprevention clinical trials with mixed results. Both metformin and aspirin activate AMPK activity but also exert pleotropic effects^21,22^. For aspirin, IC50 values derived using the MTT assay showed no clear pattern across the MCF10A-derived cell lines in our model system (Fig. 5). Similar data were noted for metformin; IC50 values were comparable across the cell lines in our model system (Fig. 6). These data suggest that, in contrast to targeted agents, the efficacy of nonspecific inhibitors such as metformin and aspirin is independent of underlying pathway aberrations. Of note, the doses of both aspirin and metformin required to achieve any reduction in cell growth were extremely high, with IC50s in the millimolar range, suggesting poor overall efficacy. Such high concentrations of drugs are impossible to achieve in humans and are thus not clinically relevant (peak concentrations of 26.8 µM aspirin in blood plasma^23^ and 14.29 µM metformin in serum have previously been reported^24^). At such high concentrations (in mM range as observed here), any growth-inhibitory effects observed are likely due to nonspecific off-target effects that are independent of endogenous target expression or target inhibition by the drugs.

## Discussion

Our finding that there is proteomic heterogeneity within the precancerous breast challenges the general assumption of a relatively homogenous preneoplastic state and underscores that selection of agents for breast cancer chemoprevention needs to be done within the context of the molecular stage of tumorigenic progression and underlying pathway aberrations. While some of the specific targeted agents (LY294002 and PD0325901) we tested are too toxic for consideration as chemoprevention agents, they provide proof of principle that understanding the molecular landscape of the precancerous state is especially pertinent for efficacy of small molecular inhibitors that target a specific pathway or molecule. Our in vitro data provide understanding of some of the differences in efficacy of chemoprevention reported in animal studies. For instance, we have previously demonstrated that a statin can prevent breast tumors in a transgenic mouse model of TNBC when the chemopreventive treatment is started prior to the onset of atypia lesions^17^. In contrast, Shibata and colleagues demonstrated no comparable efficacy when statin treatment was started at the age of 3 months, after atypia is already established, in the same mouse model of TNBC^25^. Taken together, these in vitro and in vivo observations underscore the need to understand the molecular alterations across the continuum of change from normal to invasive carcinoma, in order to tailor chemopreventive treatment and maximize efficacy.

While the current manuscript primarily sheds light on upstream molecular heterogeneity that leads to differential efficacy of drugs in prevention setting, we have tested if the differential efficacy observed in the precancer AT1 vs. DCIS state is due to differences in downstream pathway inhibition. Our Western blot analysis of MCF10.AT1 and MCF10.DCIS cells that were treated with LY294002 and PD0325901 show effective pathway inhibition in both cell states (Supplementary Figs. S4, S10 and S11). Therefore, these results point towards upstream molecular heterogeneity that leads to differential pathway activation at the baseline, rather than differential targeting by inhibitors, as the basis for better efficacy in precancer MCF10.AT1 cells.

Our findings suggesting that efficacy of intervention for prevention appears predicated on the underlying molecular state, which in turn is associated with histologic stage of tumorigenesis, are also supported by clinical observations of efficacy in women treated with tamoxifen. In the Breast Cancer Prevention Trial (National Surgical Adjuvant Breast and Bowel Project [NSABP] P-1 trial), women with increased risk of invasive breast cancer who were treated with tamoxifen were found to have a 49% reduction overall in risk of disease^26^. However, for the subset of women enrolled who were known to have atypia (analogous to the MCF10.AT1 cells in our model), the efficacy of tamoxifen chemoprevention was much higher, 86%. While patients with in situ carcinoma (analogous to the MCF10.DCIS cells in our model) were not enrolled in the Breast Cancer Prevention Trial, large trials examining efficacy of tamoxifen in patients with DCIS have been conducted. In the NSABP B-24 trial, the receipt of tamoxifen reduced the risk of disease recurrence by 39% as compared to placebo^27^, and in the UKANZ DCIS trial, tamoxifen reduced the risk of recurrence by 29%^26^. While comparisons across studies need to be made with caution, these clinical data suggest that, similar to the findings in our study, efficacy of agents for prevention of disease appears to vary depending on the underlying histologic—and thus likely molecular—context.

While in our model aspirin and metformin, the non-specific agents, showed equal efficacy across all stages of tumorigenic progression, their IC50 values were extremely high and not clinically achievable. This might explain why a majority of the epidemiological studies on aspirin use and reduction in breast cancer risk have not shown any benefit^28–31^. Similarly, window-of-opportunity trials for metformin as a chemopreventive agent have yielded conflicting results^32^, and the results from current clinical trials (NCT01101438 and ACTRN12610000219088) that are testing metformin use for primary and secondary prevention of breast cancer are pending.

In conclusion, our data provide strong evidence that multi-step progression to breast cancer is associated with substantial molecular heterogeneity that could affect the potential efficacy of any proposed chemopreventive agent. Our data also help provide mechanistic understanding to some of the clinical and epidemiologic findings in the area of breast cancer chemoprevention. We believe these data support the important principle that targeted agents developed for use in prevention should, as in the treatment of breast cancer, be tailored to the underlying molecular landscape in order to optimize the effectiveness of intervention.

## Supplementary Information

Supplementary Figures.

## Data Availability

All the data supporting the results are presented in the main manuscript and additional supporting files.

## References

[1] Tsimberidou AM (2012). Personalized medicine in a phase I clinical trials program: The MD Anderson Cancer Center initiative. Clin. Cancer Res..

[2] Von Hoff DD (2010). Pilot study using molecular profiling of patients' tumors to find potential targets and select treatments for their refractory cancers. J. Clin. Oncol..

[3] Bhardwaj A (2017). Regulation of miRNA-29c and its downstream pathways in preneoplastic progression of triple-negative breast cancer. Oncotarget.

[4] Bhardwaj A (2018). The isomiR-140-3p-regulated mevalonic acid pathway as a potential target for prevention of triple negative breast cancer. Breast Cancer Res..

[5] Ju, Z. *et al.* Integrative analyses of multilevel omics reveal preneoplastic breast to possess a molecular landscape that is globally shared with invasive basal-like breast cancer. *Cancers (Basel)* **12**. 10.3390/cancers12030722 (2020). 10.3390/cancers12030722 PMC7140033 32204397

[6] Brunner AL (2014). A shared transcriptional program in early breast neoplasias despite genetic and clinical distinctions. Genome Biol..

[7] Danforth DN (2018). Molecular profile of atypical hyperplasia of the breast. Breast Cancer Res. Treat..

[8] Dawson PJ, Wolman SR, Tait L, Heppner GH, Miller FR (1996). MCF10AT: A model for the evolution of cancer from proliferative breast disease. Am. J. Pathol..

[9] Miller, F. R., Santner, S. J., Tait, L. & Dawson, P. J. MCF10DCIS.com xenograft model of human comedo ductal carcinoma in situ. *J. Natl. Cancer Inst.* **92**, 1185–1186. 10.1093/jnci/92.14.1185a (2000). 10.1093/jnci/92.14.1185a 10904098

[10] Gonzalez-Angulo AM (2011). Functional proteomics can define prognosis and predict pathologic complete response in patients with breast cancer. Clin. Proteom..

[11] Ju Z (2015). Development of a robust classifier for quality control of reverse-phase protein arrays. Bioinformatics.

[12] Hu J (2007). Non-parametric quantification of protein lysate arrays. Bioinformatics.

[13] Manna SK, Zhang HJ, Yan T, Oberley LW, Aggarwal BB (1998). Overexpression of manganese superoxide dismutase suppresses tumor necrosis factor-induced apoptosis and activation of nuclear transcription factor-kappaB and activated protein-1. J. Biol. Chem..

[14] Software, G. P. *Confidence intervals of parameters", GraphPad Curve Fitting Guide*. http://www.graphpad.com/guides/prism/7/curve-fitting/index.htm?reg_standard_errors_and_confidence.htm.

[15] Bhardwaj A (2014). Suppression of Akt-mTOR pathway-a novel component of oncogene induced DNA damage response barrier in breast tumorigenesis. PLoS ONE.

[16] Wada M, Horinaka M, Yamazaki T, Katoh N, Sakai T (2014). The dual RAF/MEK inhibitor CH5126766/RO5126766 may be a potential therapy for RAS-mutated tumor cells. PLoS ONE.

[17] Bhardwaj A, Embury MD, Rojo RD, Albarracin C, Bedrosian I (2021). Efficacy of fluvastatin and aspirin for prevention of hormonally insensitive breast cancer. Breast Cancer Res. Treat..

[18] Liao JK, Laufs U (2005). Pleiotropic effects of statins. Annu. Rev. Pharmacol. Toxicol..

[19] Harris, R. E., Beebe-Donk, J., Doss, H. & Burr Doss, D. Aspirin, ibuprofen, and other non-steroidal anti-inflammatory drugs in cancer prevention: A critical review of non-selective COX-2 blockade (review). *Oncol. Rep.* **13**, 559–583 (2005). 15756426

[20] Saraei P, Asadi I, Kakar MA, Moradi-Kor N (2019). The beneficial effects of metformin on cancer prevention and therapy: A comprehensive review of recent advances. Cancer Manag. Res..

[21] Amin AR, Attur MG, Pillinger M, Abramson SB (1999). The pleiotropic functions of aspirin: mechanisms of action. Cell. Mol. Life Sci..

[22] Schulten, H. J. Pleiotropic effects of metformin on cancer. *Int. J. Mol. Sci.* **19**. 10.3390/ijms19102850 (2018). 10.3390/ijms19102850 PMC6213406 30241339

[23] Nagelschmitz J (2014). Pharmacokinetics and pharmacodynamics of acetylsalicylic acid after intravenous and oral administration to healthy volunteers. Clin. Pharmacol..

[24] Hess C, Unger M, Madea B, Stratmann B, Tschoepe D (2018). Range of therapeutic metformin concentrations in clinical blood samples and comparison to a forensic case with death due to lactic acidosis. Forensic Sci. Int..

[25] Shibata MA (2003). Comparative effects of lovastatin on mammary and prostate oncogenesis in transgenic mouse models. Carcinogenesis.

[26] Fisher B (1998). Tamoxifen for prevention of breast cancer: report of the National Surgical Adjuvant Breast and Bowel Project P-1 Study. J. Natl. Cancer Inst..

[27] Mamounas EP (2003). NSABP breast cancer clinical trials: Recent results and future directions. Clin. Med. Res..

[28] Tsoi KKF, Ho JMW, Chan FCH, Sung JJY (2019). Long-term use of low-dose aspirin for cancer prevention: A 10-year population cohort study in Hong Kong. Int. J. Cancer.

[29] Bens A (2018). Low-dose aspirin use and risk of contralateral breast cancer: A Danish nationwide cohort study. Prev. Med..

[30] Dierssen-Sotos T (2016). Use of non-steroidal anti-inflammatory drugs and risk of breast cancer: The Spanish Multi-Case-control (MCC) study. BMC Cancer.

[31] Lu L, Shi L, Zeng J, Wen Z (2017). Aspirin as a potential modality for the chemoprevention of breast cancer: A dose-response meta-analysis of cohort studies from 857,831 participants. Oncotarget.

[32] Jones VC, Dietze EC, Jovanovic-Talisman T, McCune JS, Seewaldt VL (2020). Metformin and chemoprevention: Potential for heart-healthy targeting of biologically aggressive breast cancer. Front Public Health.

