# Supplementary material for: The molecular heterogeneity of the precancerous breast affects drug efficacy
Source: Sci Rep. 2022 Jul 22;12:12590. doi: 10.1038/s41598-022-16779-y (PMC9307756; doi:10.1038/s41598-022-16779-y)
Supplement: Supplementary file 1 — Supplementary Figures. [file 41598_2022_16779_MOESM1_ESM.pptx]

## Slide 1
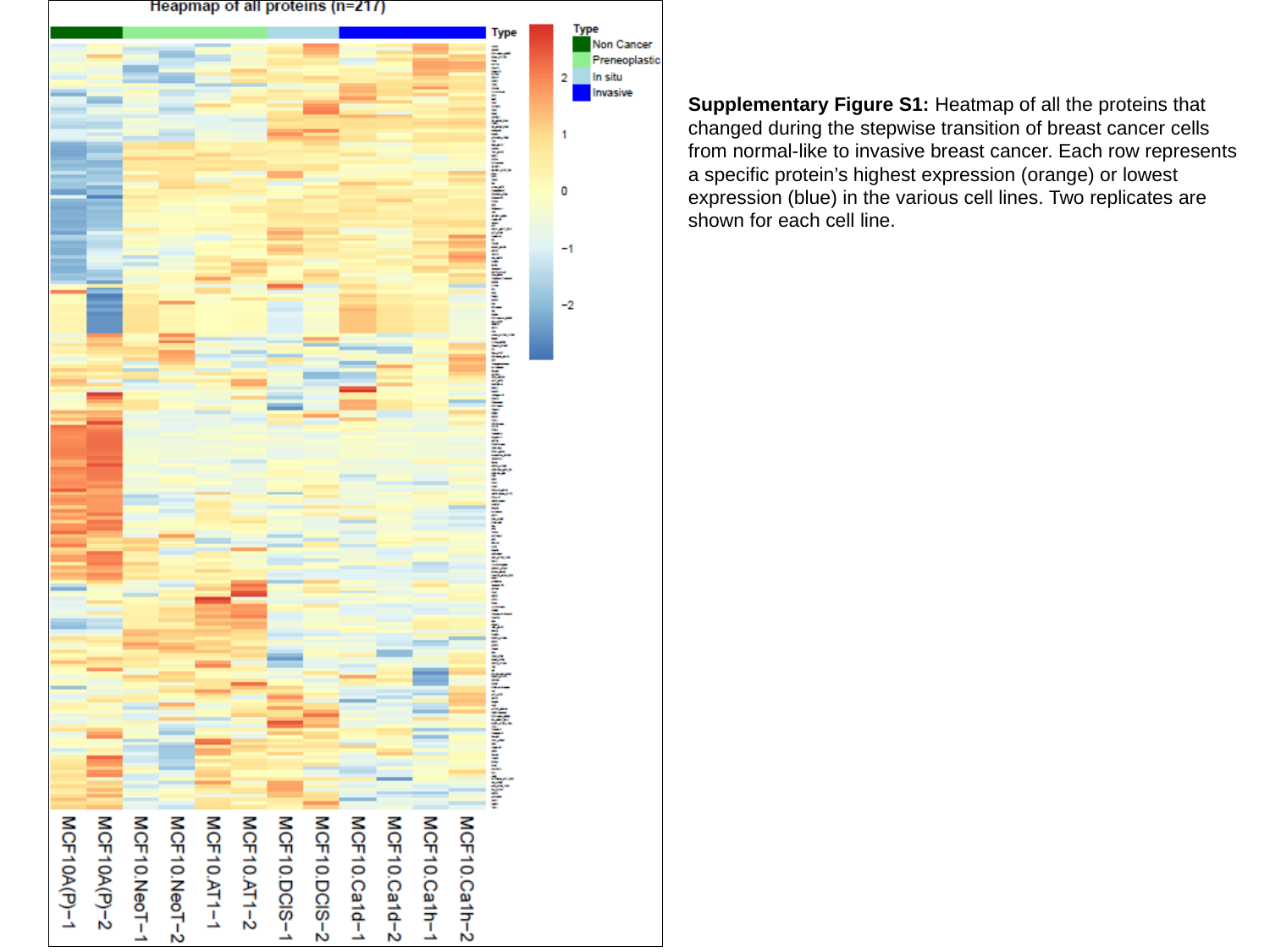

Supplementary Figure S1: Heatmap of all the proteins that changed during the stepwise transition of breast cancer cells from normal-like to invasive breast cancer. Each row represents a specific protein’s highest expression (orange) or lowest expression (blue) in the various cell lines. Two replicates are shown for each cell line.

## Slide 2
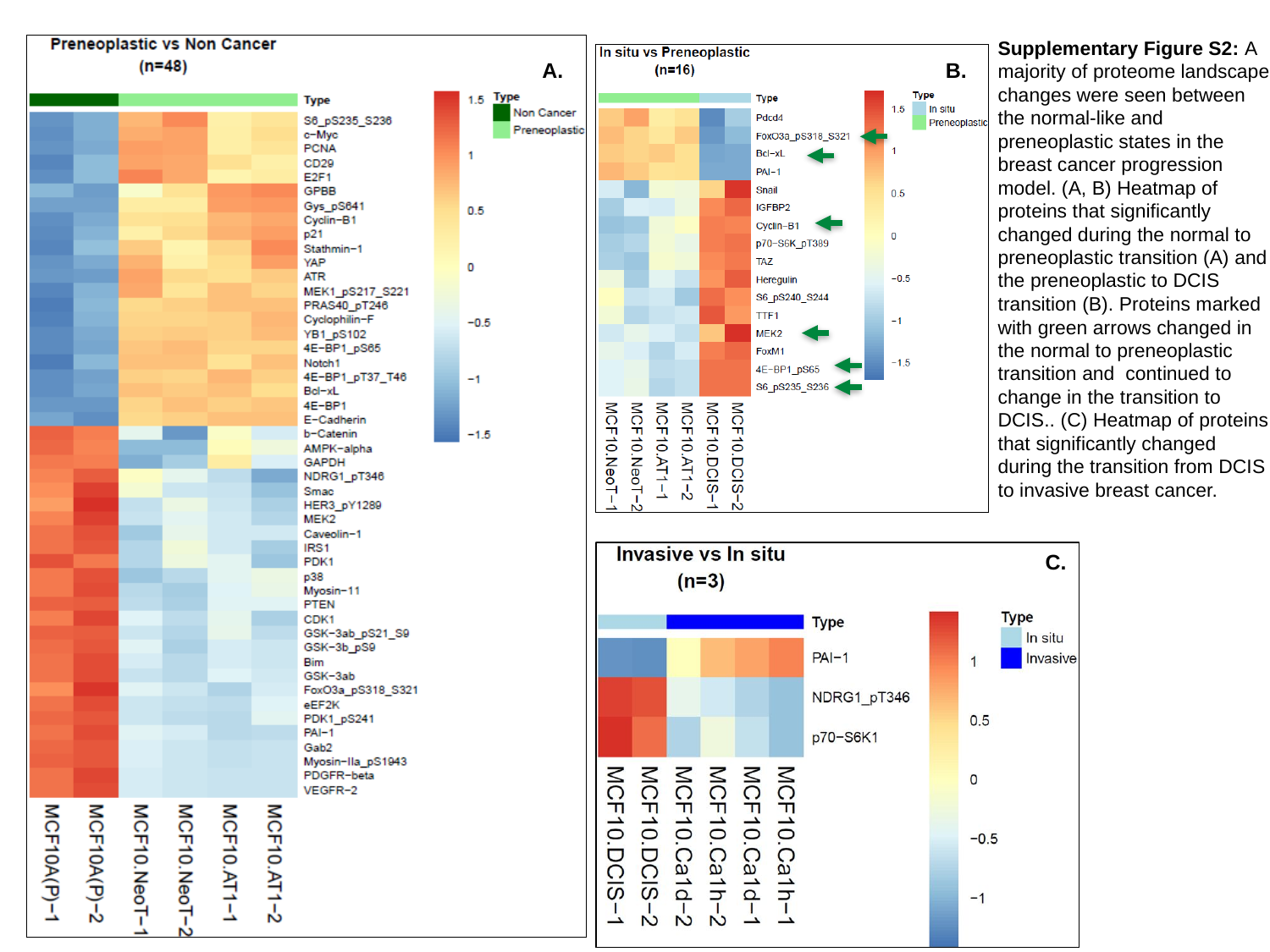

Supplementary Figure S2: A majority of proteome landscape changes were seen between the normal-like and preneoplastic states in the breast cancer progression model. (A, B) Heatmap of proteins that significantly changed during the normal to preneoplastic transition (A) and the preneoplastic to DCIS transition (B). Proteins marked with green arrows changed in the normal to preneoplastic transition and continued to change in the transition to DCIS.. (C) Heatmap of proteins that significantly changed during the transition from DCIS to invasive breast cancer.
A.
B.
C.

## Slide 3
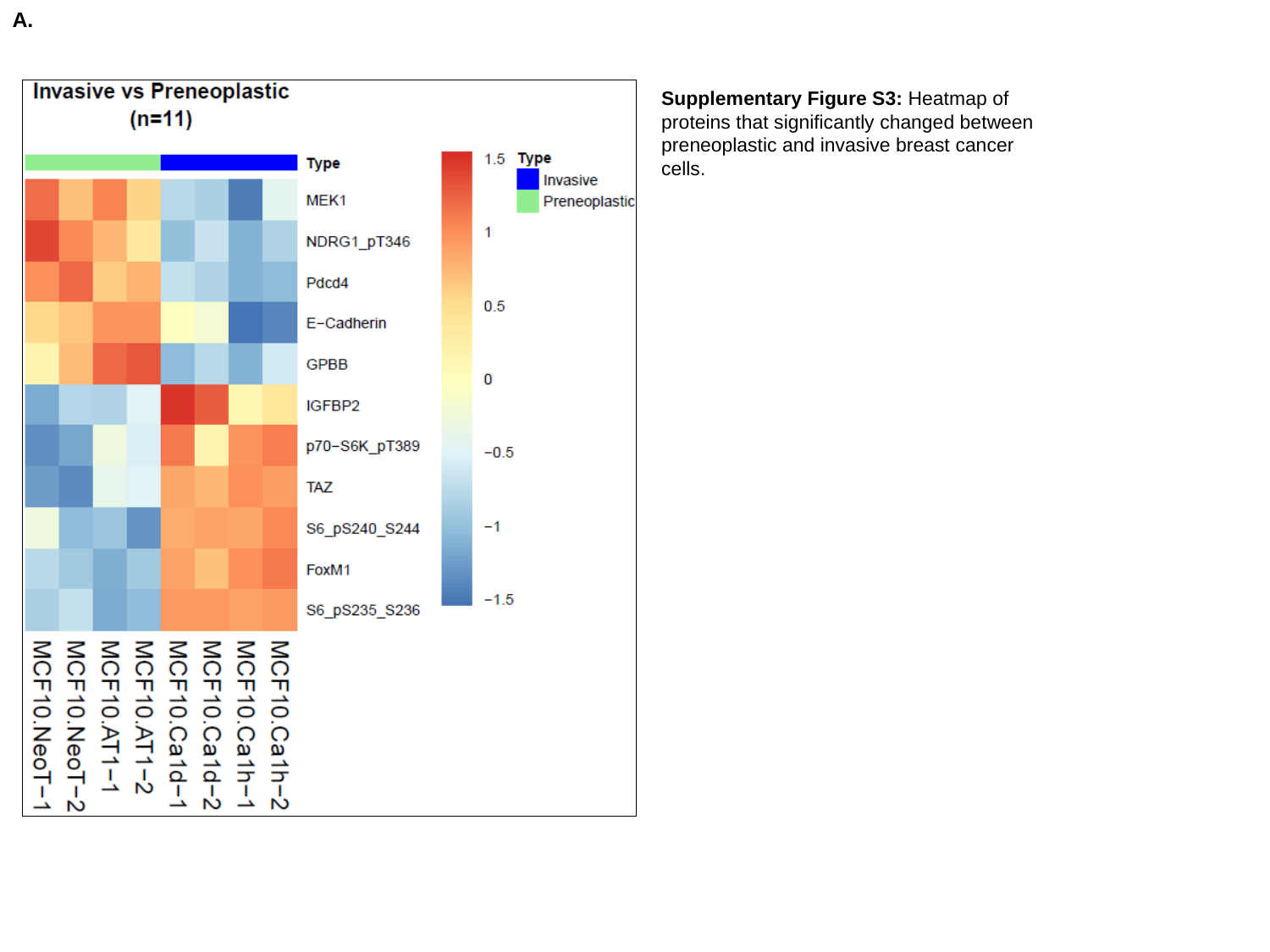

A.
Supplementary Figure S3: Heatmap of proteins that significantly changed between preneoplastic and invasive breast cancer cells.

## Slide 4
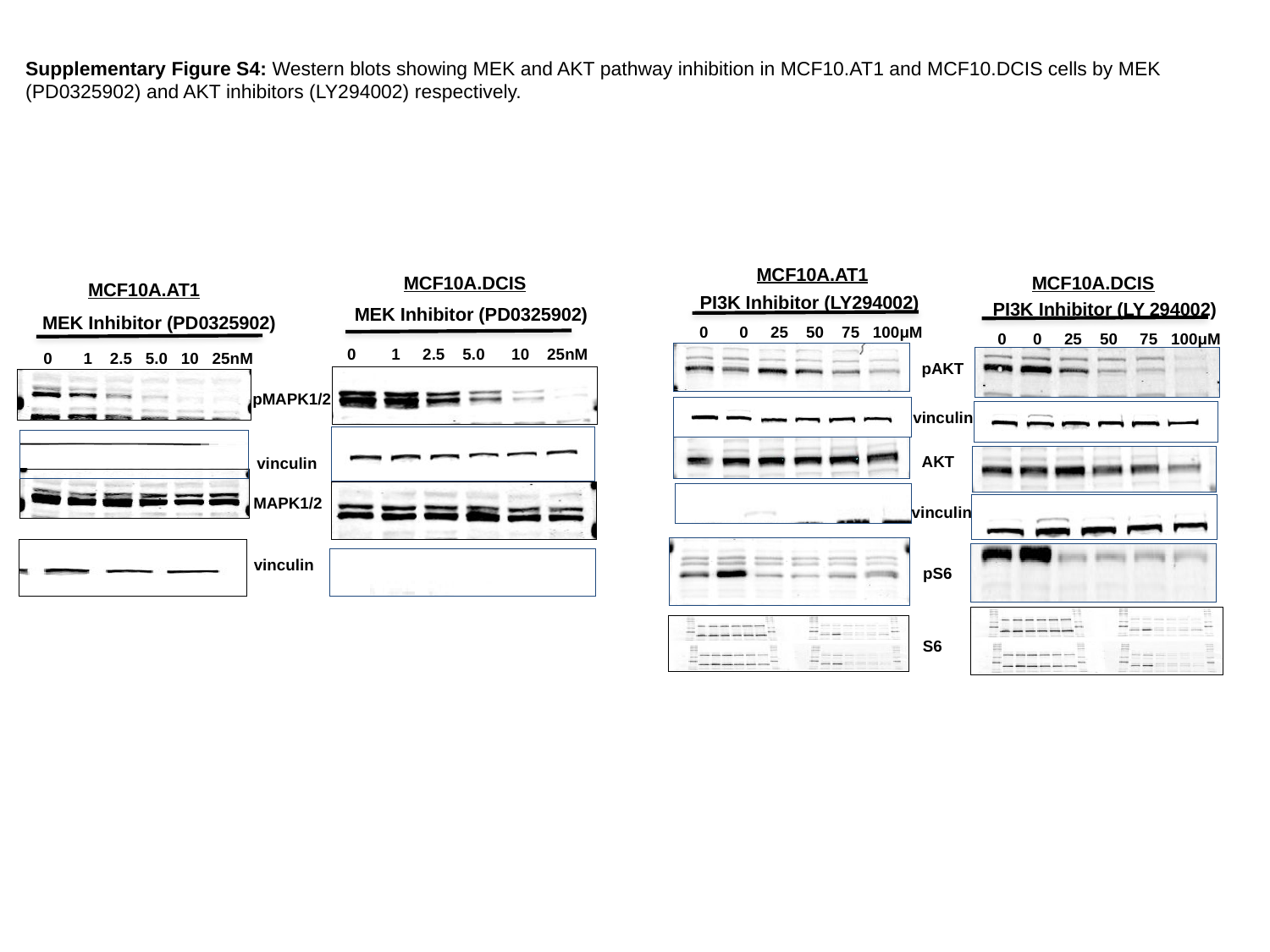

Supplementary Figure S4: Western blots showing MEK and AKT pathway inhibition in MCF10.AT1 and MCF10.DCIS cells by MEK (PD0325902) and AKT inhibitors (LY294002) respectively.
MCF10A.AT1
MCF10A.DCIS
MCF10A.DCIS
MCF10A.AT1
PI3K Inhibitor (LY294002)
PI3K Inhibitor (LY 294002)
MEK Inhibitor (PD0325902)
MEK Inhibitor (PD0325902)
 0 0 25 50 75 100μM
 0 0 25 50 75 100μM
 0 1 2.5 5.0 10 25nM
 0 1 2.5 5.0 10 25nM
pAKT
pMAPK1/2
vinculin
AKT
vinculin
MAPK1/2
vinculin
vinculin
pS6
S6

## Slide 5
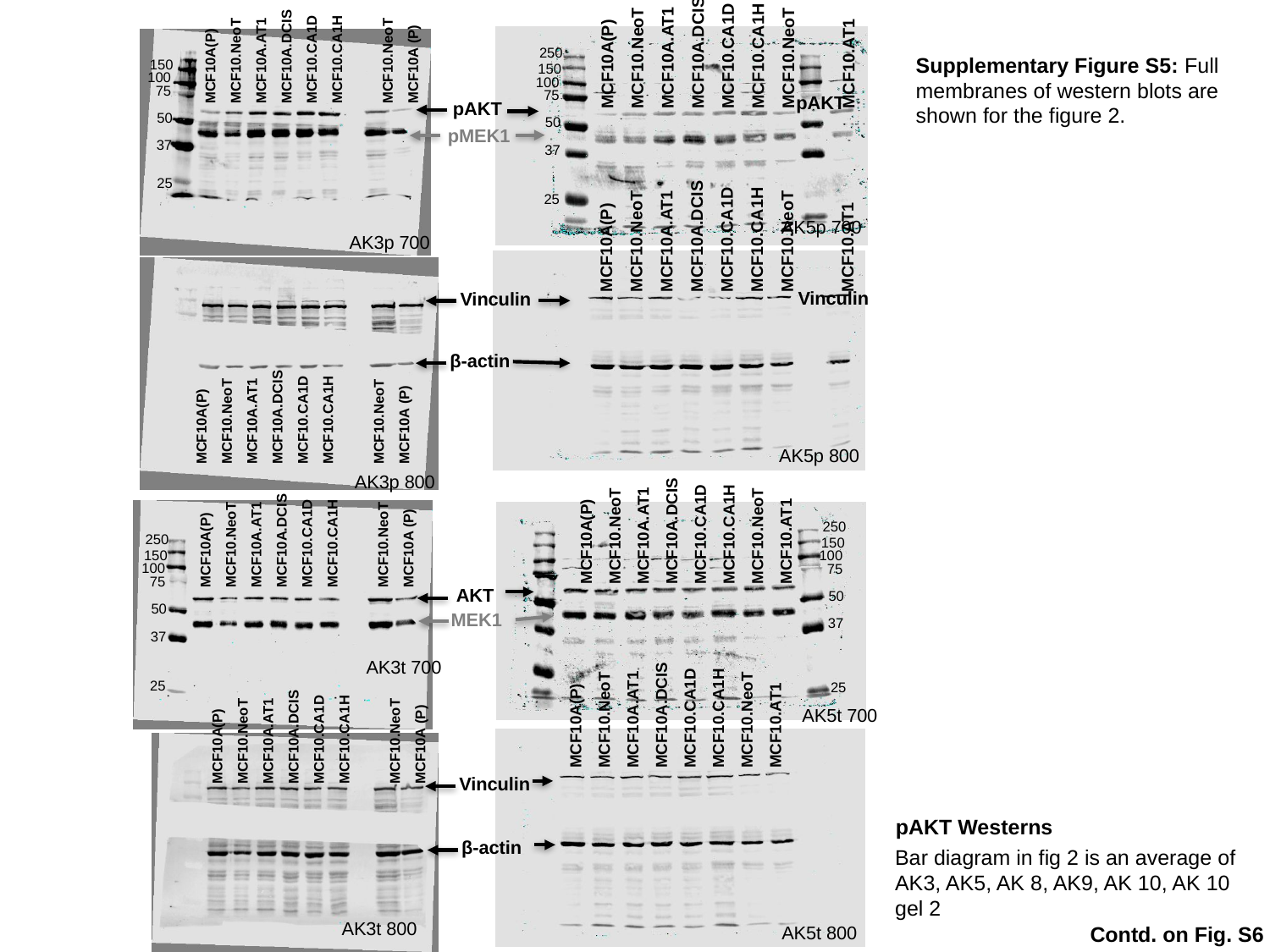

MCF10A(P)
MCF10.NeoT
MCF10A.AT1
MCF10A.DCIS
MCF10.CA1D
MCF10.CA1H
MCF10.NeoT
MCF10.AT1
MCF10A(P)
MCF10.NeoT
MCF10A.AT1
MCF10A.DCIS
MCF10.CA1D
MCF10.CA1H
MCF10.NeoT
MCF10A (P)
250
Supplementary Figure S5: Full membranes of western blots are shown for the figure 2.
150
150
100
100
75
75
pAKT
pAKT
MCF10A(P)
MCF10.NeoT
MCF10A.AT1
MCF10A.DCIS
MCF10.CA1D
MCF10.CA1H
MCF10.NeoT
MCF10.AT1
50
50
pMEK1
37
37
25
25
AK5p 700
AK3p 700
Vinculin
Vinculin
MCF10A(P)
MCF10.NeoT
MCF10A.AT1
MCF10A.DCIS
MCF10.CA1D
MCF10.CA1H
MCF10.NeoT
MCF10A (P)
β-actin
MCF10A(P)
MCF10.NeoT
MCF10A.AT1
MCF10A.DCIS
MCF10.CA1D
MCF10.CA1H
MCF10.NeoT
MCF10A (P)
MCF10A(P)
MCF10.NeoT
MCF10A.AT1
MCF10A.DCIS
MCF10.CA1D
MCF10.CA1H
MCF10.NeoT
MCF10.AT1
AK5p 800
AK3p 800
250
250
150
150
100
100
75
75
AKT
50
50
MCF10A(P)
MCF10.NeoT
MCF10A.AT1
MCF10A.DCIS
MCF10.CA1D
MCF10.CA1H
MCF10.NeoT
MCF10.AT1
MEK1
MCF10A(P)
MCF10.NeoT
MCF10A.AT1
MCF10A.DCIS
MCF10.CA1D
MCF10.CA1H
MCF10.NeoT
MCF10A (P)
37
37
AK3t 700
25
25
AK5t 700
Vinculin
pAKT Westerns
β-actin
Bar diagram in fig 2 is an average of AK3, AK5, AK 8, AK9, AK 10, AK 10 gel 2
AK3t 800
AK5t 800
Contd. on Fig. S6

## Slide 6
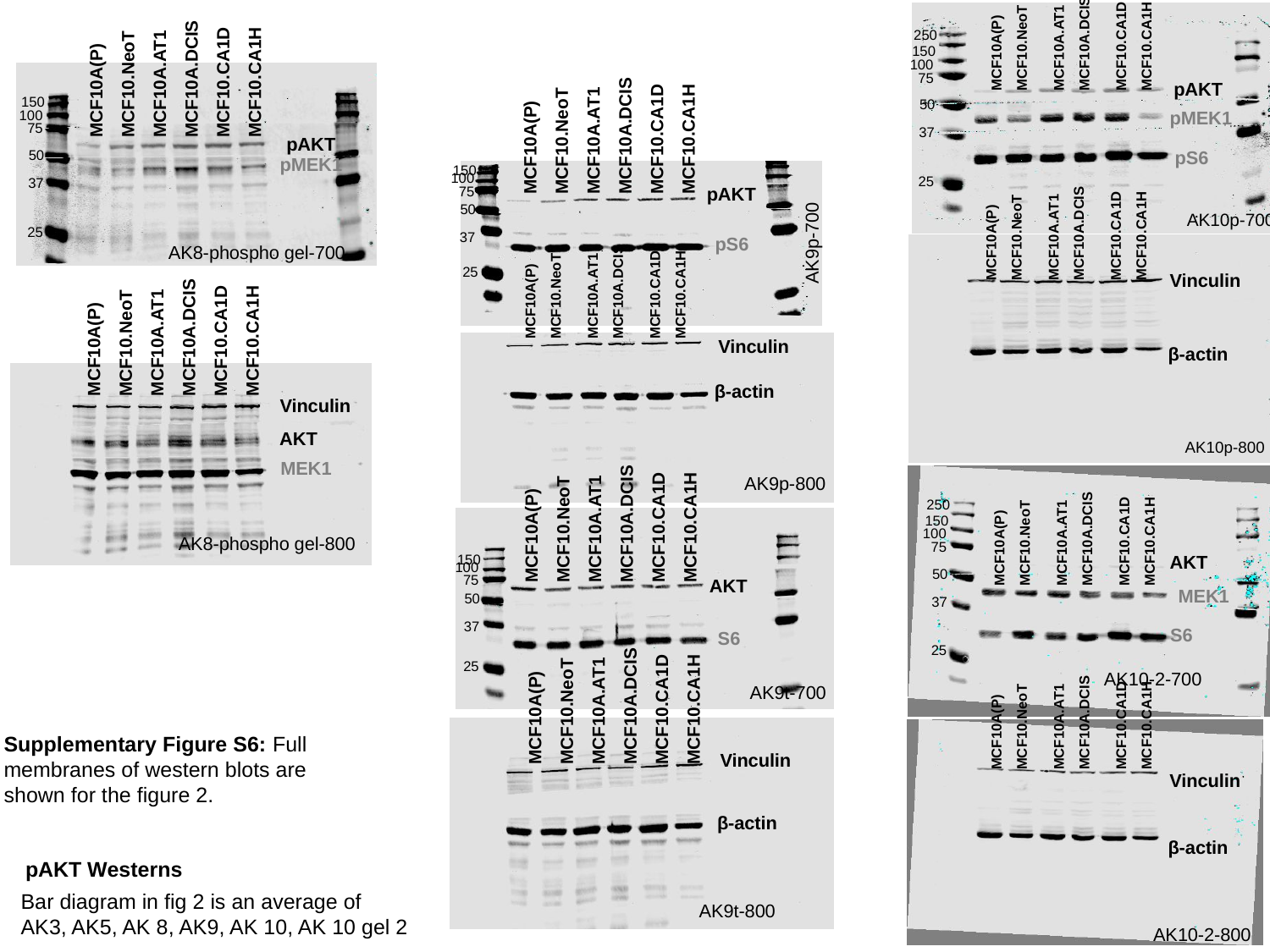

MCF10A(P)
MCF10.NeoT
MCF10A.AT1
MCF10A.DCIS
MCF10.CA1D
MCF10.CA1H
MCF10A(P)
MCF10.NeoT
MCF10A.AT1
MCF10A.DCIS
MCF10.CA1D
MCF10.CA1H
250
MCF10A(P)
MCF10.NeoT
MCF10A.AT1
MCF10A.DCIS
MCF10.CA1D
MCF10.CA1H
150
100
75
pAKT
150
50
pMEK1
100
75
37
pAKT
MCF10A(P)
MCF10.NeoT
MCF10A.AT1
MCF10A.DCIS
MCF10.CA1D
MCF10.CA1H
50
pS6
pMEK1
150
100
25
37
pAKT
75
50
MCF10A(P)
MCF10.NeoT
MCF10A.AT1
MCF10A.DCIS
MCF10.CA1D
MCF10.CA1H
AK10p-700
AK9p-700
25
37
pS6
AK8-phospho gel-700
MCF10A(P)
MCF10.NeoT
MCF10A.AT1
MCF10A.DCIS
MCF10.CA1D
MCF10.CA1H
25
Vinculin
Vinculin
β-actin
β-actin
Vinculin
AKT
MCF10A(P)
MCF10.NeoT
MCF10A.AT1
MCF10A.DCIS
MCF10.CA1D
MCF10.CA1H
AK10p-800
MCF10A(P)
MCF10.NeoT
MCF10A.AT1
MCF10A.DCIS
MCF10.CA1D
MCF10.CA1H
MEK1
AK9p-800
250
150
100
AK8-phospho gel-800
75
150
AKT
100
50
75
AKT
MEK1
50
37
MCF10A(P)
MCF10.NeoT
MCF10A.AT1
MCF10A.DCIS
MCF10.CA1D
MCF10.CA1H
37
S6
S6
MCF10A(P)
MCF10.NeoT
MCF10A.AT1
MCF10A.DCIS
MCF10.CA1D
MCF10.CA1H
25
25
AK10-2-700
AK9t-700
Supplementary Figure S6: Full membranes of western blots are shown for the figure 2.
Vinculin
Vinculin
β-actin
β-actin
pAKT Westerns
Bar diagram in fig 2 is an average of AK3, AK5, AK 8, AK9, AK 10, AK 10 gel 2
AK9t-800
AK10-2-800

## Slide 7
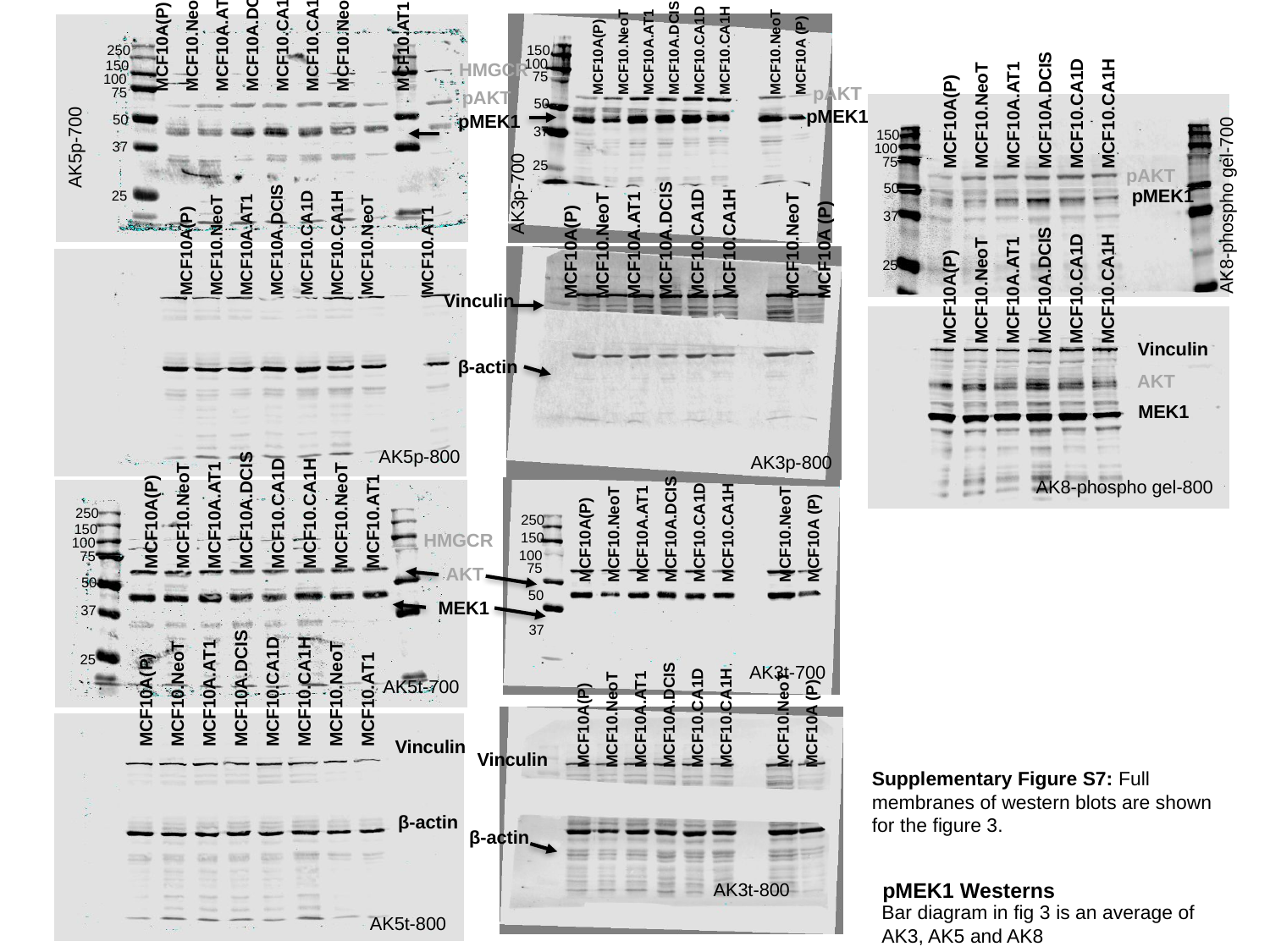

MCF10A(P)
MCF10.NeoT
MCF10A.AT1
MCF10A.DCIS
MCF10.CA1D
MCF10.CA1H
MCF10.NeoT
MCF10.AT1
MCF10A(P)
MCF10.NeoT
MCF10A.AT1
MCF10A.DCIS
MCF10.CA1D
MCF10.CA1H
MCF10.NeoT
MCF10A (P)
A.
MCF10A(P)
MCF10.NeoT
MCF10A.AT1
MCF10A.DCIS
MCF10.CA1D
MCF10.CA1H
250
150
100
150
HMGCR
75
100
MCF10A(P)
MCF10.NeoT
MCF10A.AT1
MCF10A.DCIS
MCF10.CA1D
MCF10.CA1H
MCF10.NeoT
MCF10A (P)
pAKT
75
pAKT
50
pMEK1
MCF10A(P)
MCF10.NeoT
MCF10A.AT1
MCF10A.DCIS
MCF10.CA1D
MCF10.CA1H
MCF10.NeoT
MCF10.AT1
pMEK1
50
37
150
AK5p-700
37
100
75
25
pAKT
50
AK3p-700
pMEK1
AK8-phospho gel-700
25
MCF10A(P)
MCF10.NeoT
MCF10A.AT1
MCF10A.DCIS
MCF10.CA1D
MCF10.CA1H
37
25
Vinculin
Vinculin
β-actin
AKT
MCF10A(P)
MCF10.NeoT
MCF10A.AT1
MCF10A.DCIS
MCF10.CA1D
MCF10.CA1H
MCF10.NeoT
MCF10.AT1
MCF10A(P)
MCF10.NeoT
MCF10A.AT1
MCF10A.DCIS
MCF10.CA1D
MCF10.CA1H
MCF10.NeoT
MCF10A (P)
MEK1
AK5p-800
AK3p-800
AK8-phospho gel-800
250
250
150
150
HMGCR
100
100
75
75
MCF10A(P)
MCF10.NeoT
MCF10A.AT1
MCF10A.DCIS
MCF10.CA1D
MCF10.CA1H
MCF10.NeoT
MCF10.AT1
AKT
MCF10A(P)
MCF10.NeoT
MCF10A.AT1
MCF10A.DCIS
MCF10.CA1D
MCF10.CA1H
MCF10.NeoT
MCF10A (P)
50
50
MEK1
37
37
25
AK3t-700
AK5t-700
Vinculin
Vinculin
Supplementary Figure S7: Full membranes of western blots are shown for the figure 3.
β-actin
β-actin
pMEK1 Westerns
AK3t-800
Bar diagram in fig 3 is an average of AK3, AK5 and AK8
AK5t-800
AK5t-800

## Slide 8
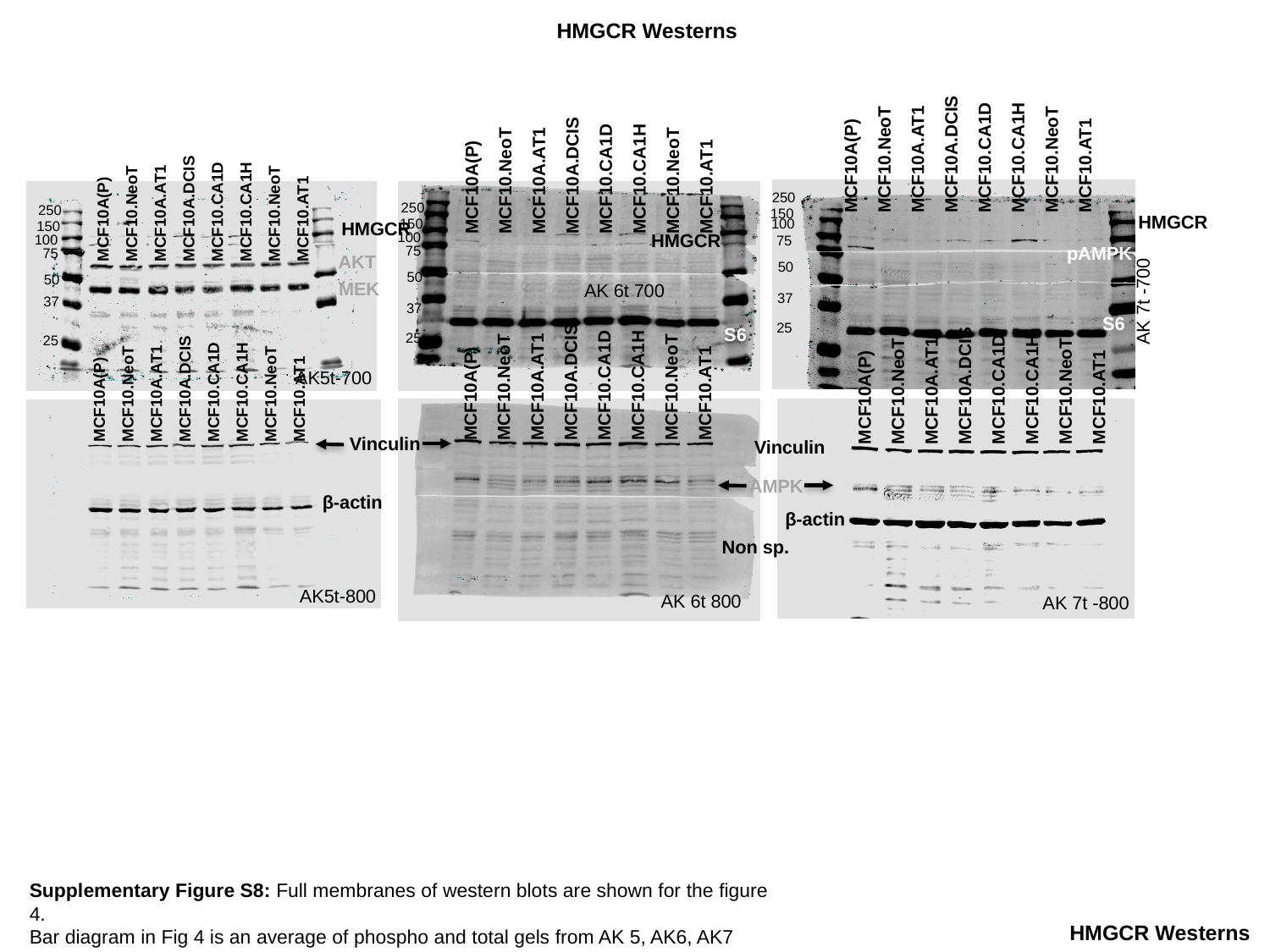

HMGCR Westerns
MCF10A(P)
MCF10.NeoT
MCF10A.AT1
MCF10A.DCIS
MCF10.CA1D
MCF10.CA1H
MCF10.NeoT
MCF10.AT1
MCF10A(P)
MCF10.NeoT
MCF10A.AT1
MCF10A.DCIS
MCF10.CA1D
MCF10.CA1H
MCF10.NeoT
MCF10.AT1
MCF10A(P)
MCF10.NeoT
MCF10A.AT1
MCF10A.DCIS
MCF10.CA1D
MCF10.CA1H
MCF10.NeoT
MCF10.AT1
250
250
250
150
HMGCR
150
100
150
HMGCR
100
HMGCR
100
75
75
pAMPK
75
MCF10A(P)
MCF10.NeoT
MCF10A.AT1
MCF10A.DCIS
MCF10.CA1D
MCF10.CA1H
MCF10.NeoT
MCF10.AT1
AKT
MCF10A(P)
MCF10.NeoT
MCF10A.AT1
MCF10A.DCIS
MCF10.CA1D
MCF10.CA1H
MCF10.NeoT
MCF10.AT1
50
50
50
MCF10A(P)
MCF10.NeoT
MCF10A.AT1
MCF10A.DCIS
MCF10.CA1D
MCF10.CA1H
MCF10.NeoT
MCF10.AT1
MEK
AK 6t 700
AK 7t -700
37
37
37
S6
25
S6
25
25
AK5t-700
Vinculin
Vinculin
AMPK
β-actin
β-actin
Non sp.
AK5t-800
AK 6t 800
AK 7t -800
Supplementary Figure S8: Full membranes of western blots are shown for the figure 4.
Bar diagram in Fig 4 is an average of phospho and total gels from AK 5, AK6, AK7
HMGCR Westerns

## Slide 9
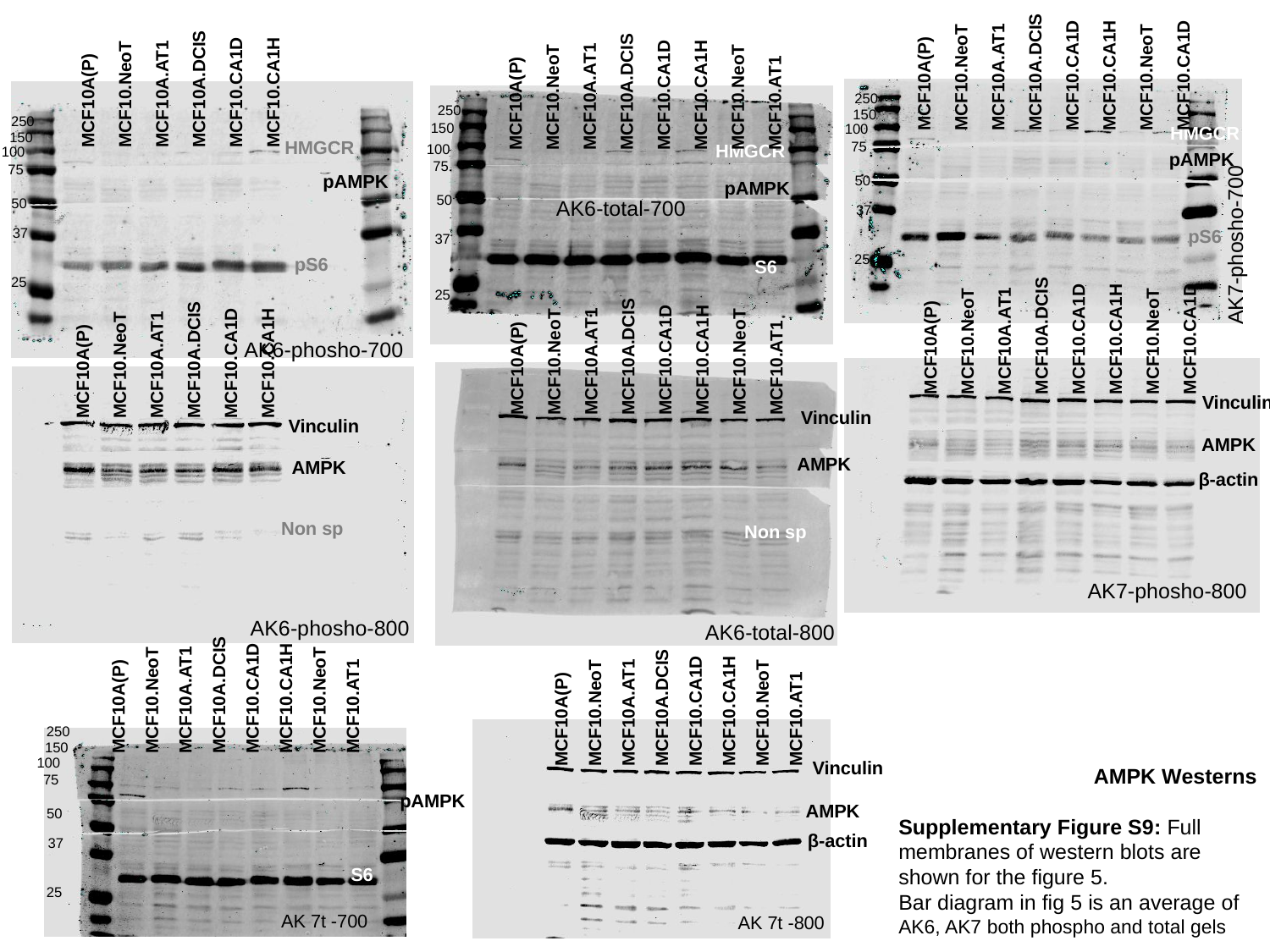

MCF10A(P)
MCF10.NeoT
MCF10A.AT1
MCF10A.DCIS
MCF10.CA1D
MCF10.CA1H
MCF10.NeoT
MCF10.CA1D
MCF10A(P)
MCF10.NeoT
MCF10A.AT1
MCF10A.DCIS
MCF10.CA1D
MCF10.CA1H
MCF10.NeoT
MCF10.AT1
MCF10A(P)
MCF10.NeoT
MCF10A.AT1
MCF10A.DCIS
MCF10.CA1D
MCF10.CA1H
250
250
150
250
150
100
HMGCR
150
HMGCR
75
HMGCR
100
100
pAMPK
75
75
pAMPK
50
pAMPK
MCF10A(P)
MCF10.NeoT
MCF10A.AT1
MCF10A.DCIS
MCF10.CA1D
MCF10.CA1H
MCF10.NeoT
MCF10.CA1D
50
50
AK6-total-700
37
MCF10A(P)
MCF10.NeoT
MCF10A.AT1
MCF10A.DCIS
MCF10.CA1D
MCF10.CA1H
MCF10.NeoT
MCF10.AT1
37
pS6
37
AK7-phosho-700
MCF10A(P)
MCF10.NeoT
MCF10A.AT1
MCF10A.DCIS
MCF10.CA1D
MCF10.CA1H
25
pS6
S6
25
25
AK6-phosho-700
Vinculin
Vinculin
Vinculin
AMPK
AMPK
AMPK
β-actin
Non sp
Non sp
MCF10A(P)
MCF10.NeoT
MCF10A.AT1
MCF10A.DCIS
MCF10.CA1D
MCF10.CA1H
MCF10.NeoT
MCF10.AT1
MCF10A(P)
MCF10.NeoT
MCF10A.AT1
MCF10A.DCIS
MCF10.CA1D
MCF10.CA1H
MCF10.NeoT
MCF10.AT1
AK7-phosho-800
AK6-phosho-800
AK6-total-800
250
150
100
Vinculin
AMPK Westerns
75
pAMPK
AMPK
50
Supplementary Figure S9: Full membranes of western blots are shown for the figure 5.
Bar diagram in fig 5 is an average of AK6, AK7 both phospho and total gels
β-actin
37
S6
25
AK 7t -700
AK 7t -800

## Slide 10
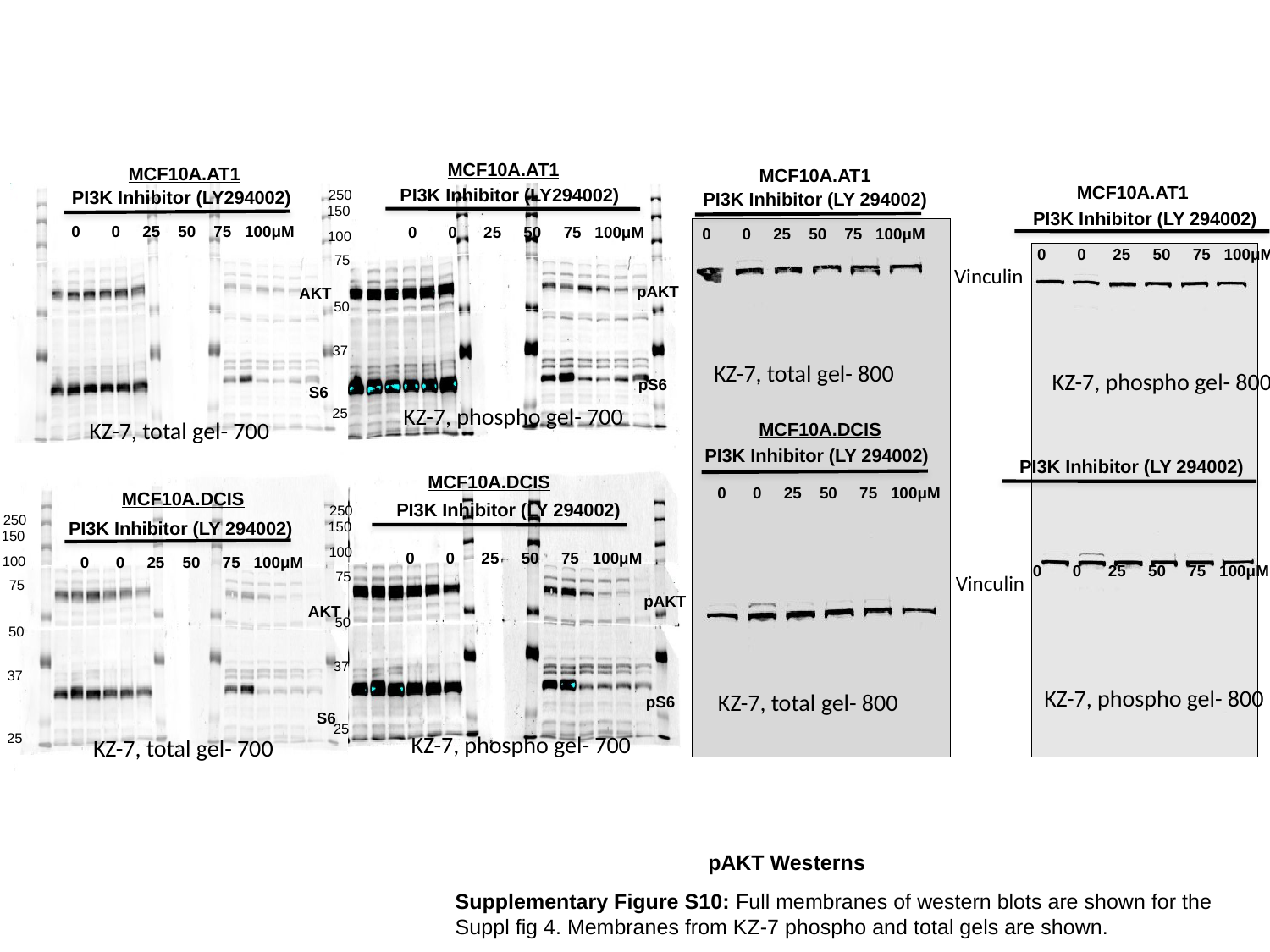

MCF10A.AT1
MCF10A.AT1
MCF10A.AT1
MCF10A.AT1
PI3K Inhibitor (LY294002)
PI3K Inhibitor (LY294002)
250
PI3K Inhibitor (LY 294002)
150
PI3K Inhibitor (LY 294002)
 0 0 25 50 75 100μM
 0 0 25 50 75 100μM
 0 0 25 50 75 100μM
100
 0 0 25 50 75 100μM
75
Vinculin
pAKT
AKT
50
37
KZ-7, total gel- 800
KZ-7, phospho gel- 800
pS6
S6
KZ-7, phospho gel- 700
25
KZ-7, total gel- 700
MCF10A.DCIS
PI3K Inhibitor (LY 294002)
PI3K Inhibitor (LY 294002)
MCF10A.DCIS
 0 0 25 50 75 100μM
MCF10A.DCIS
PI3K Inhibitor (LY 294002)
250
250
PI3K Inhibitor (LY 294002)
150
150
100
 0 0 25 50 75 100μM
 0 0 25 50 75 100μM
100
 0 0 25 50 75 100μM
75
Vinculin
75
pAKT
AKT
50
50
37
37
KZ-7, phospho gel- 800
KZ-7, total gel- 800
pS6
S6
25
25
KZ-7, phospho gel- 700
KZ-7, total gel- 700
pAKT Westerns
Supplementary Figure S10: Full membranes of western blots are shown for the Suppl fig 4. Membranes from KZ-7 phospho and total gels are shown.

## Slide 11
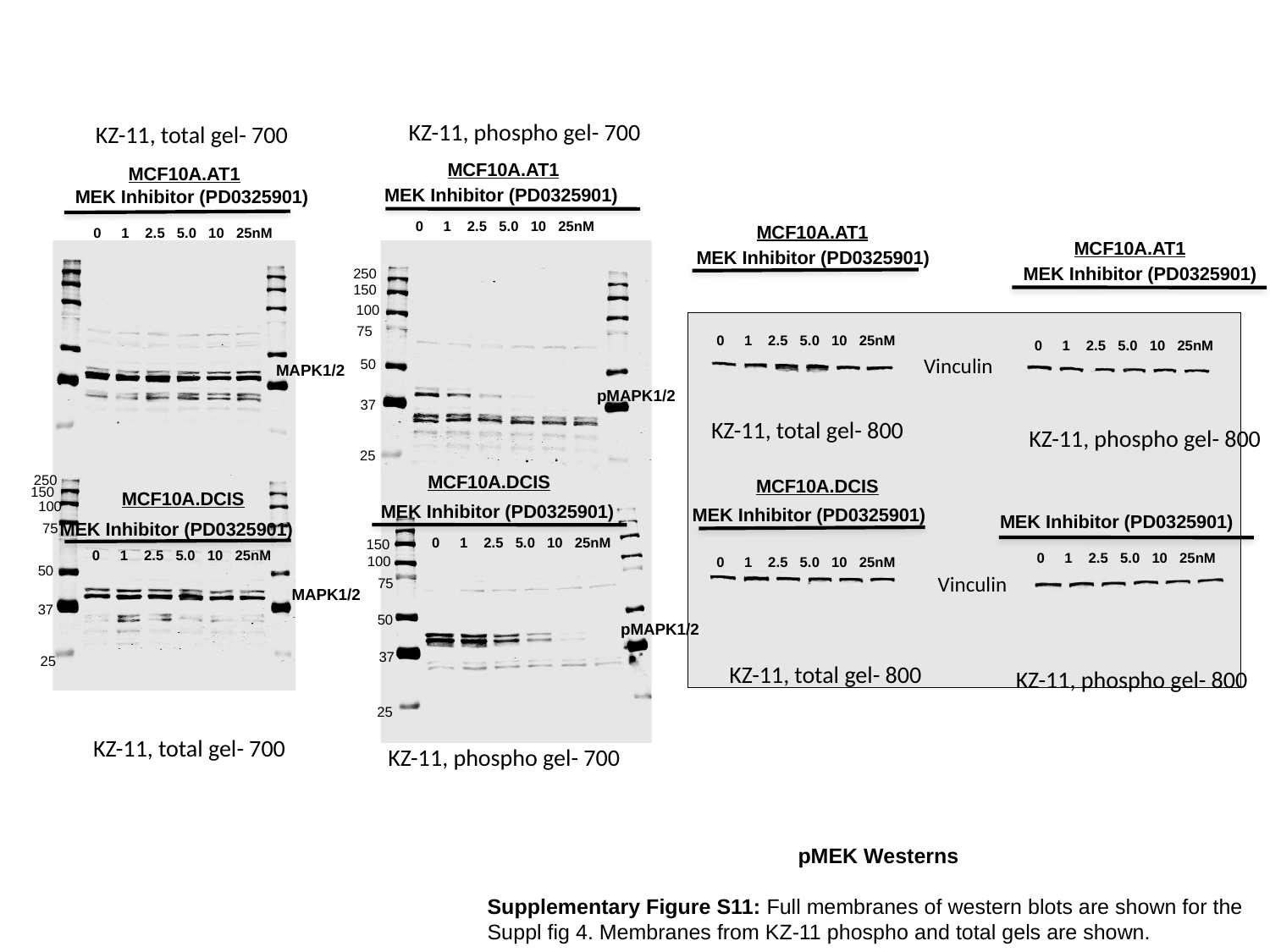

KZ-11, phospho gel- 700
KZ-11, total gel- 700
MCF10A.AT1
MCF10A.AT1
MEK Inhibitor (PD0325901)
MEK Inhibitor (PD0325901)
 0 1 2.5 5.0 10 25nM
MCF10A.AT1
 0 1 2.5 5.0 10 25nM
MCF10A.AT1
MEK Inhibitor (PD0325901)
MEK Inhibitor (PD0325901)
250
150
100
75
 0 1 2.5 5.0 10 25nM
 0 1 2.5 5.0 10 25nM
Vinculin
50
MAPK1/2
pMAPK1/2
37
KZ-11, total gel- 800
KZ-11, phospho gel- 800
25
MCF10A.DCIS
250
MCF10A.DCIS
150
MCF10A.DCIS
100
 MEK Inhibitor (PD0325901)
MEK Inhibitor (PD0325901)
MEK Inhibitor (PD0325901)
MEK Inhibitor (PD0325901)
75
 0 1 2.5 5.0 10 25nM
150
 0 1 2.5 5.0 10 25nM
 0 1 2.5 5.0 10 25nM
100
 0 1 2.5 5.0 10 25nM
50
Vinculin
75
MAPK1/2
37
50
pMAPK1/2
37
25
KZ-11, total gel- 800
KZ-11, phospho gel- 800
25
KZ-11, total gel- 700
KZ-11, phospho gel- 700
pMEK Westerns
Supplementary Figure S11: Full membranes of western blots are shown for the Suppl fig 4. Membranes from KZ-11 phospho and total gels are shown.

## Slide 12
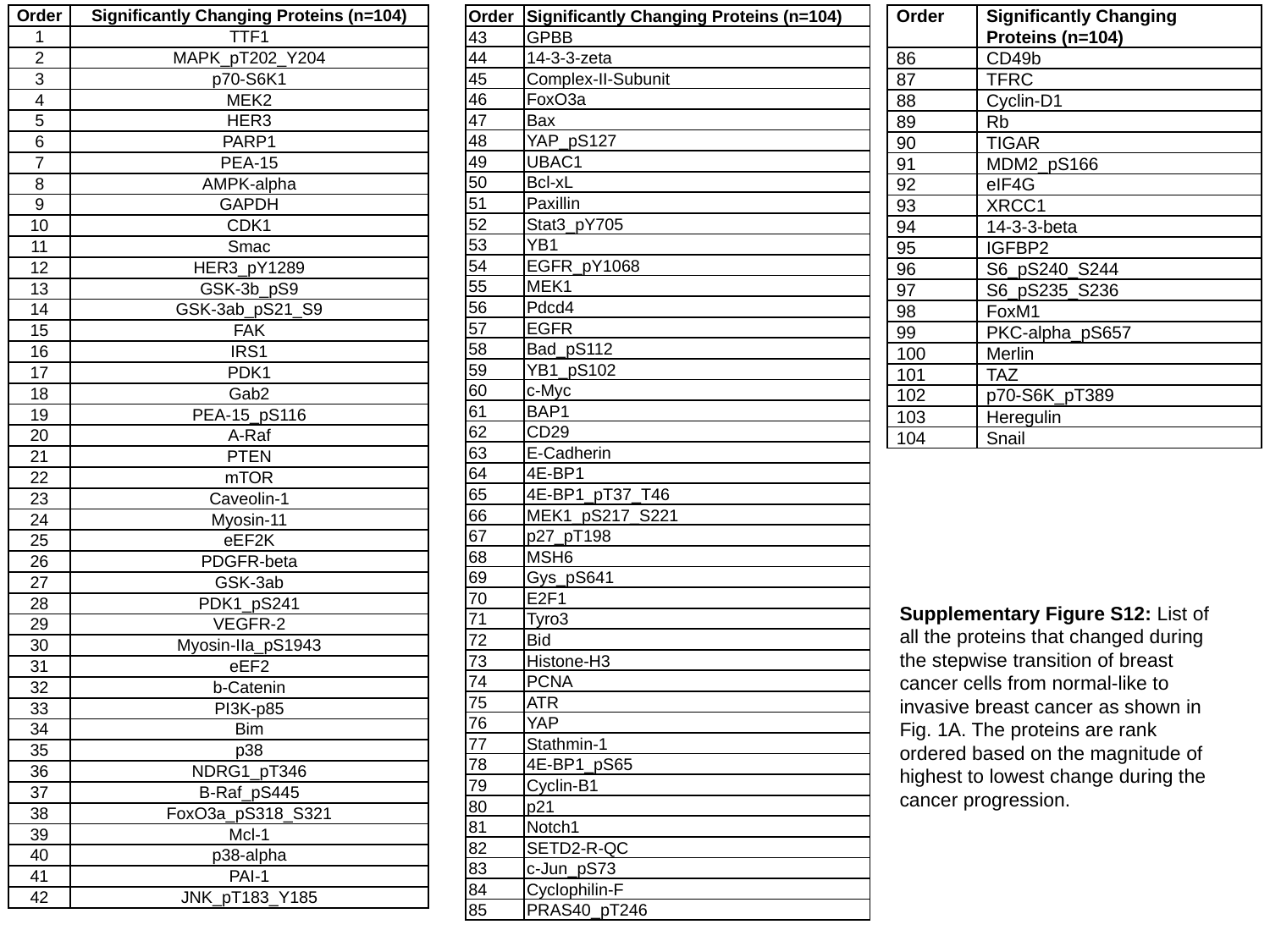

| Order | Significantly Changing Proteins (n=104) |
| --- | --- |
| 1 | TTF1 |
| 2 | MAPK\_pT202\_Y204 |
| 3 | p70-S6K1 |
| 4 | MEK2 |
| 5 | HER3 |
| 6 | PARP1 |
| 7 | PEA-15 |
| 8 | AMPK-alpha |
| 9 | GAPDH |
| 10 | CDK1 |
| 11 | Smac |
| 12 | HER3\_pY1289 |
| 13 | GSK-3b\_pS9 |
| 14 | GSK-3ab\_pS21\_S9 |
| 15 | FAK |
| 16 | IRS1 |
| 17 | PDK1 |
| 18 | Gab2 |
| 19 | PEA-15\_pS116 |
| 20 | A-Raf |
| 21 | PTEN |
| 22 | mTOR |
| 23 | Caveolin-1 |
| 24 | Myosin-11 |
| 25 | eEF2K |
| 26 | PDGFR-beta |
| 27 | GSK-3ab |
| 28 | PDK1\_pS241 |
| 29 | VEGFR-2 |
| 30 | Myosin-IIa\_pS1943 |
| 31 | eEF2 |
| 32 | b-Catenin |
| 33 | PI3K-p85 |
| 34 | Bim |
| 35 | p38 |
| 36 | NDRG1\_pT346 |
| 37 | B-Raf\_pS445 |
| 38 | FoxO3a\_pS318\_S321 |
| 39 | Mcl-1 |
| 40 | p38-alpha |
| 41 | PAI-1 |
| 42 | JNK\_pT183\_Y185 |
| Order | Significantly Changing Proteins (n=104) |
| --- | --- |
| 43 | GPBB |
| 44 | 14-3-3-zeta |
| 45 | Complex-II-Subunit |
| 46 | FoxO3a |
| 47 | Bax |
| 48 | YAP\_pS127 |
| 49 | UBAC1 |
| 50 | Bcl-xL |
| 51 | Paxillin |
| 52 | Stat3\_pY705 |
| 53 | YB1 |
| 54 | EGFR\_pY1068 |
| 55 | MEK1 |
| 56 | Pdcd4 |
| 57 | EGFR |
| 58 | Bad\_pS112 |
| 59 | YB1\_pS102 |
| 60 | c-Myc |
| 61 | BAP1 |
| 62 | CD29 |
| 63 | E-Cadherin |
| 64 | 4E-BP1 |
| 65 | 4E-BP1\_pT37\_T46 |
| 66 | MEK1\_pS217\_S221 |
| 67 | p27\_pT198 |
| 68 | MSH6 |
| 69 | Gys\_pS641 |
| 70 | E2F1 |
| 71 | Tyro3 |
| 72 | Bid |
| 73 | Histone-H3 |
| 74 | PCNA |
| 75 | ATR |
| 76 | YAP |
| 77 | Stathmin-1 |
| 78 | 4E-BP1\_pS65 |
| 79 | Cyclin-B1 |
| 80 | p21 |
| 81 | Notch1 |
| 82 | SETD2-R-QC |
| 83 | c-Jun\_pS73 |
| 84 | Cyclophilin-F |
| 85 | PRAS40\_pT246 |
| Order | Significantly Changing Proteins (n=104) |
| --- | --- |
| 86 | CD49b |
| 87 | TFRC |
| 88 | Cyclin-D1 |
| 89 | Rb |
| 90 | TIGAR |
| 91 | MDM2\_pS166 |
| 92 | eIF4G |
| 93 | XRCC1 |
| 94 | 14-3-3-beta |
| 95 | IGFBP2 |
| 96 | S6\_pS240\_S244 |
| 97 | S6\_pS235\_S236 |
| 98 | FoxM1 |
| 99 | PKC-alpha\_pS657 |
| 100 | Merlin |
| 101 | TAZ |
| 102 | p70-S6K\_pT389 |
| 103 | Heregulin |
| 104 | Snail |
Supplementary Figure S12: List of all the proteins that changed during the stepwise transition of breast cancer cells from normal-like to invasive breast cancer as shown in Fig. 1A. The proteins are rank ordered based on the magnitude of highest to lowest change during the cancer progression.
